# Molecular reshaping of phage-displayed Interleukin-2 at beta chain receptor interface to obtain potent super-agonists with improved developability profiles

**DOI:** 10.1038/s42003-023-05188-0

**Published:** 2023-08-09

**Authors:** Gertrudis Rojas, Ernesto Relova-Hernández, Annia Pérez-Riverón, Camila Castro-Martínez, Osmany Diaz-Bravo, Yanelys Cabrera Infante, Tania Gómez, Joaquín Solozábal, Ana Beatriz DíazBravo, Maren Schubert, Marlies Becker, Beatriz Pérez-Massón, Dayana Pérez-Martínez, Rydell Alvarez-Arzola, Osmany Guirola, Glay Chinea, Luis Graca, Stefan Dübel, Kalet León, Tania Carmenate

**Affiliations:** 1 Center of Molecular Immunology, calle 216 esq 15, apartado 16040, Atabey, Playa, CP 11300 La Habana, Cuba; 2 Technische Universität Braunschweig, Institute of Biochemistry, Biotechnology and Bioinformatics, Department of Biotechnology, Spielmannstraße 7, 38106 Braunschweig, Germany; 3 Center of Genetic Engineering and Biotechnology, Ave 31 e/ 158 y 190, apartado 6162, Playa, CP 11300 La Habana, Cuba; 4 Instituto de Medicina Molecular João Lobo Antunes, Faculdade de Medicina da Universidade de Lisboa, Centro Académico de Medicina de Lisboa, Lisbon, Portugal

**Keywords:** Interleukins, Combinatorial libraries, Protein design

## Abstract

Interleukin-2 (IL-2) engineered versions, with biased immunological functions, have emerged from yeast display and rational design. Here we reshaped the human IL-2 interface with the IL-2 receptor beta chain through the screening of phage-displayed libraries. Multiple beta super-binders were obtained, having increased receptor binding ability and improved developability profiles. Selected variants exhibit an accumulation of negatively charged residues at the interface, which provides a better electrostatic complementarity to the beta chain, and faster association kinetics. These findings point to mechanistic differences with the already reported superkines, characterized by a conformational switch due to the rearrangement of the hydrophobic core. The molecular bases of the favourable developability profile were tracked to a single residue: L92. Recombinant Fc-fusion proteins including our variants are superior to those based on H9 superkine in terms of expression levels in mammalian cells, aggregation resistance, stability, in vivo enhancement of immune effector responses, and anti-tumour effect.

## Abstract (web summary)

Engineered interleukin-2 (IL-2) variants yield better electrostatic complementarity to the IL-2 receptor beta chain and faster association kinetics leading to in vivo enhancement of immune effector responses and anti-tumor effects.

## Introduction

Dual immunological effects of Interleukin-2 (IL-2), a potent stimulator of effector responses and the key cytokine keeping self-tolerance through the activation of T regulatory cells (Tregs)^1^, have theoretical and practical implications. Structural studies^2,3^ support a model of IL-2/receptor interactions that explains this fine functional balance. While effector T and natural killer (NK) cells express a moderate affinity dimeric IL-2 receptor (IL-2R), formed by beta (also known as CD122) and gamma (CD132) subunits, Tregs display constitutively a trimeric high affinity receptor incorporating alpha chain (CD25)^4^. Therefore, in physiological conditions, Tregs have the advantage to use the limiting amounts of IL-2 and exert their down-modulatory activity. Upon strong immune stimulation, IL-2 production is enough to sustain the activation and function of effector cells which produce more IL-2, resulting in a positive feedback loop. Activated effector T cells express transiently the alpha receptor subunit, which increases their sensitivity to IL-2 in a temporarily regulated fashion. Over-activation of effector T cells through IL-2R results in activation-induced cell death, preventing uncontrolled and potentially dangerous immune responses beyond the resolution of the original damage^5^.

Despite such fine-tuning of permanent self-tolerance and acute/memory immune responses, there are pathological conditions in which disrupting the natural balance and reinforcing a particular IL-2 function would be desirable. This is the case of cancer, as immune responses to self (or slightly modified) tumour cells are usually not powerful enough to control the disease. Enhancement of immune effector functions with high-dose IL-2 has a long history of therapeutic success in a subset of patients^6^, although high toxicity^7^ and undesired Treg expansion^8^ have limited its usefulness. Protein engineering allows creating a functional bias through direct IL-2 modification. Disruption of human IL-2 (hIL-2) alpha chain binding abrogates its preferential use by Tregs, resulting in potent anti-tumour responses^9^. A different approach is based on hIL-2 variants with increased binding ability to the beta chain, which do not depend on the alpha chain for high affinity engagement, and are thus strong stimulators of effector cells. H9 superkine, a molecule obtained from directed evolution through yeast display, is the most representative member of this class^10^.

The introduction of mutations in engineered cytokines often results in poor pharmaceutical developability, as changes modulating receptor interactions can increase the intrinsic aggregation propensity and instability of tightly regulated bioactive proteins which naturally do not reach high concentrations and long persistence in the body^11^. Modifying such non-optimal scaffolds to obtain the desired functions, together with high expression levels and enhanced stability, is thus challenging. Recently, fully in silico IL-2 engineering, based on stabilizing protein core structure rather than interfaces, gave rise to a new wave of thermostable superkines without any experimental optimization^12^. A revolutionary strategy relied on de novo design of highly stable artificial proteins able to bind the dimeric IL-2 receptor through a combination of in silico techniques and yeast display^13^. Despite the attractiveness of this approach, the use of totally new proteins as drugs could result in undesired interactions in the body.

The current study was aimed at optimizing hIL-2 interface with CD122, using filamentous phage-based directed evolution. This approach, previously unexplored, identified diverse structural solutions, showing the importance of both surface electrostatic interactions and hydrophobic core rearrangements in the modulation of IL-2/IL-2R beta interaction. Unlike in variants emerging from yeast display^10^, improved CD122 binding was accompanied by a favourable developability profile, characterized by high expression levels, low aggregation propensity, and high stability. These properties were translated into improved in vivo results in terms of biased stimulation of immune effector cells and anti-tumour activity. The molecular bases of drastic impairments/improvements in developability were tracked to amino acid (aa) 92. Our results showed the feasibility of reshaping IL-2 interface with CD122 to obtain potent super-agonists with an appealing therapeutic potential. The phage display platform showed once again its usefulness to minimize biophysical liabilities associated with cytokine modification^14^.

## Results

### Soft-randomization of phage-displayed hIL-2 beta chain interface revealed hotspots modulating the interaction

**Fig. 1:**
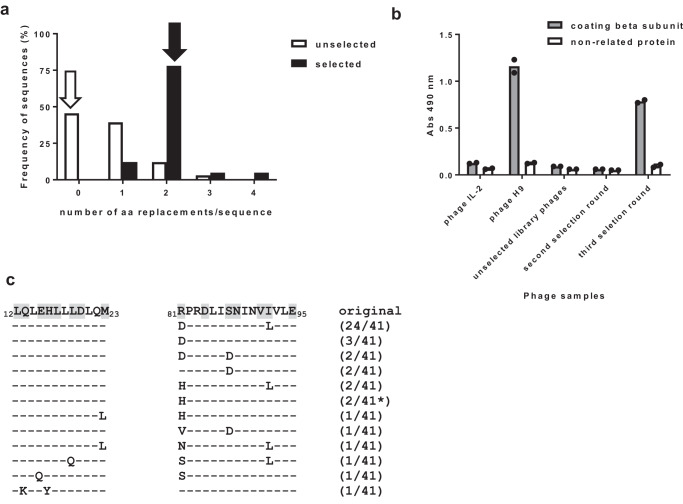
Phage selection from a soft-randomization human IL-2 library diversified at the beta chain interface. The library was constructed by Kunkel mutagenesis on a hIL-2 gene-containing phagemid template, using two mutagenic spiked oligonucleotides which targeted beta interface segments 12–23 and 81–95. The composition of oligonucleotides was biased towards the original wild-type (wt) sequence, but introduced limited variability at eight and seven codons of these segments, respectively. Library phages were panned on the immobilized extracellular domain (ECD) of hIL-2 receptor beta chain (three selection rounds). Deducing protein sequences of a sample of cytokine-displaying clones picked before and after selection revealed that the number of mutations per sequence increased upon selection (**a**). White and black arrows indicate the most frequent number of aa replacements in the library and in the selected phage population, respectively. Beta chain reactivity of phage pools (10^11^ viral particles/mL) obtained before and after selection rounds was evaluated by ELISA on polyvinyl chloride microtitration plates coated with beta chain ECD (**b**). Bound phages were detected with an anti-M13 antibody conjugated to horseradish peroxidase. Phage-displayed versions of hIL-2 and H9 superkine were used as controls. Binding specificity was assessed with a non-related coating protein (bovine serum albumin). Each phage sample was evaluated twice on each coating protein. Bars indicate the mean absorbance values of both determinations, while dots represent individual data points. Sequence analysis of selected hIL-2 variants identified aa replacements within the targeted segments (**c**). The original wt sequences appear in the first line, with diversified residues shaded in grey. Replacing aa are shown in the selected sequences below. Short lines indicate the conservation of the original residue. The numbers between parentheses represent the frequency of each sequence within the sample of selected clones. The asterisk indicates the presence of additional mutations outside of the targeted regions.

Soft-randomization was chosen for an initial mutational scanning at hIL-2/hIL-2R beta interface, focused on solvent-exposed (>20%) hIL-2 residues close to the beta chain (<5 Å). A library of 7 × 10^7^ members was obtained by Kunkel mutagenesis^15^ with spiked mutagenic oligonucleotides targeting hIL-2 segments 12–23 and 81–95. Oligonucleotide composition was biased towards the original sequence, but introduced limited variability at codons coding for eight and seven amino acids within these segments, respectively. The analysis of 33 cytokine-displaying library clones revealed the abundance of the original wild-type (wt) hIL-2 sequence (45.5%), probably due to limited mutagenesis efficiency on a non-mutated hIL-2 template (Fig. 1a). There were 18 unique mutated sequences (1–3 aa replacements each). Single-mutated variants predominated among them (Fig. 1a). The wt residue was most represented at all targeted positions, confirming soft randomization without gross library construction artefacts. The library size and the frequency of single-mutated variants (39.4%) guaranteed a comprehensive scanning of individual mutations (285 in 15 targeted positions). Double-mutated variants (37 905 combinations) could also be fully explored within the library clones displaying two simultaneous aa changes (12.1%). Coverage of more complex combinations of replacements was likely to be very limited.

Phage selection on the immobilized recombinant extracellular domain (ECD) of hIL-2R beta subunit resulted in the enrichment of a library subpopulation. Non-mutated hIL-2 was not detected among 41 selected clones, which exhibited 1–4 mutations (Fig. 1a). Reactivity to CD122 ECD increased along the selection procedure (Fig. 1b). Inspection of selected sequences (Fig. 1c) showed the predominance of the double-mutated variant containing R81D + I92L. Changes at positions 81 (diverse replacing aa) and 92 (always I92L) were recurrently found among other selected variants. S87D was also repeated. Three hotspots (positions 81, 87, 92) potentially modulating IL-2 interaction with CD122 were thus identified. Mutations in the segment 12–23 were less frequent, and did not show a regular pattern, indicating lower optimization potential (Fig. 1c). Only three selected clones displayed replacements at non-targeted locations (seven unexpected mutations). K35Q, found twice, was known to be highly selectable due to an increase in display levels^14^.

### Combinatorial mutagenesis scanning around the hotspots underscored complex sequence patterns associated with increased beta chain reactivity

**Fig. 2:**
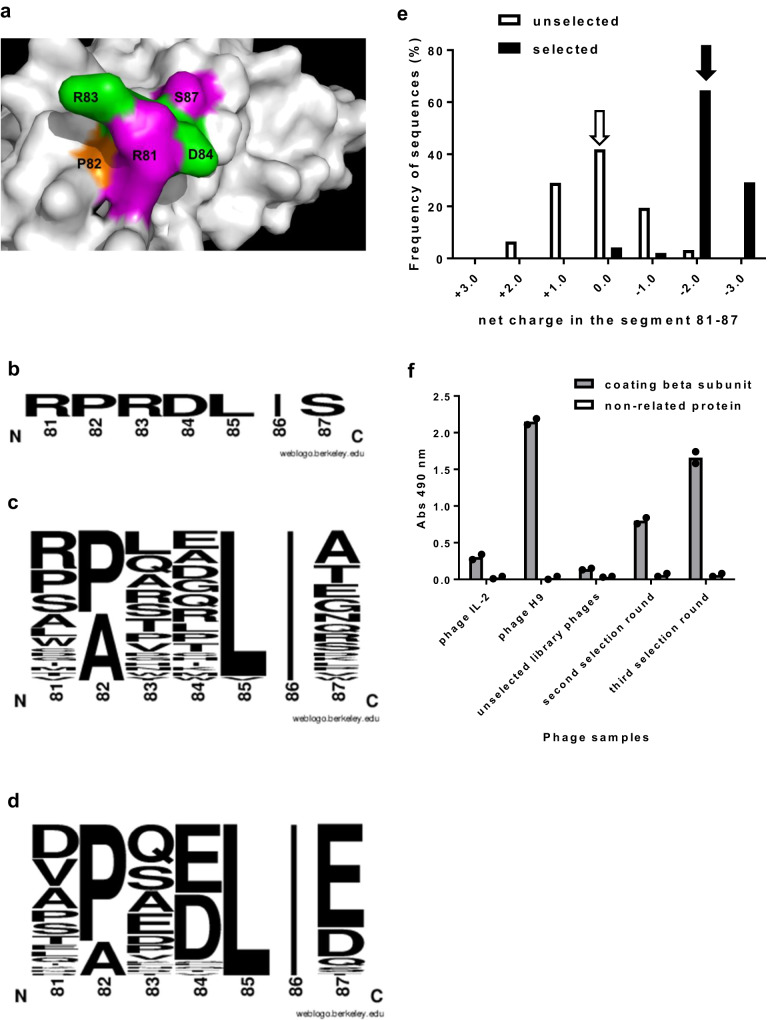
Phage selection from the secondary library S1. Library design is illustrated in (**a**). Human IL-2 structure (from PDB code 2B5I) is represented as a grey surface generated with Pymol. R81 and S87 (solvent-exposed mutational hotspots identified in the initial screening) are highlighted in magenta. Neighbour protruding residues (R83, D84) are shown in green. The adjacent P82 appears in orange. The library was constructed by Kunkel mutagenesis on a hIL-2 gene-containing phagemid template, using a mutagenic oligonucleotide that introduced full randomization at positions 81, 83, 84 and 87, and limited variability (Pro/Ala) at position 82. Library phages were panned on the immobilized extracellular domain (ECD) of hIL-2 receptor beta chain (three rounds). The original non-mutated sequence of hIL-2 segment 81–87 is shown in (**b**). Web logos (**c**, **d**) represent diversity along the primary sequence. The size of each letter is proportional to the frequency of the corresponding amino acid at a given position. Sequence profiles of samples of non-selected cytokine-displaying S1 library clones (**c**), and of selected clones (**d**), are shown. Analysis of the net charge in segment 81–87 revealed a shift towards higher negative charges upon selection (**e**). White and black arrows indicate the most frequent net charge before and after selection, respectively. CD122 reactivity of phage pools (10^11^ viral particles/mL) obtained before and after selection rounds was evaluated by ELISA on polyvinyl chloride microtitration plates coated with beta chain ECD (**f**). Bound phages were detected with an anti-M13 antibody conjugated to horseradish peroxidase Phage-displayed hIL-2 and H9 superkine were used as controls. Binding specificity was assessed with a non-related coating protein (bovine serum albumin). Each phage sample was evaluated twice on each coating protein. Bars indicate the mean absorbance values of both determinations, while dots represent individual data points.

The secondary library S1 was totally randomized at positions 81 and 87 (identified as hotspots), as well as at neighbour residues 83 and 84. The side chains of the four aa at these locations form a highly protruding cluster in the 3D structure (Fig. 2a). P82, although not solvent-exposed, is adjacent to the targeted residues and could influence their orientations. Position 82 was occupied by a mixture of Pro and Ala, to add structural variability to the library. Sequencing of 39 cytokine-displaying library clones showed the non-mutated hIL-2 template sequence in 20.5% of them (Fig. 2b), and 31 unique mutated sequences. Their analysis confirmed the expected diversity pattern (Fig. 2c). The frequency of fortuitous changes was very low, L85V replacement in one clone and a single aa deletion in another one (Fig. 2c). Sequences outside the targeted region remained unchanged. S1 library size (1.2 × 10^9^ clones) was enough to explore combinatorial diversity within the targeted segment.

Sequence analysis of 59 clones selected on beta chain ECD revealed a vast diversity, with 48 unique sequences (none repeated more than three times). However, shared features were identified among them (Fig. 2d). Arg residues 81 and 83 were replaced by diverse aa, including negatively charged residues (Asp/Glu). D84 tended to be either conserved or substituted by Glu. S87 was recurrently replaced by either Glu or Asp. This mutation pattern suggested a role for electrostatic interactions in optimizing binding. Fig. 2e shows the remarkable charge shift in segment 81–87 upon selection. Whereas hIL-2 has a positive net charge in this region (+1) and neutral 81–87 segments predominate in the unselected library, almost all selected clones exhibited a net negative charge (mostly −2 and −3). The selected phage pool showed increased beta chain reactivity, while low background levels against bovine serum albumin (a non-related coating molecule) ruled out the possibility of massive non-specific selection of promiscuous binders (Fig. 2f).

**Fig. 3:**
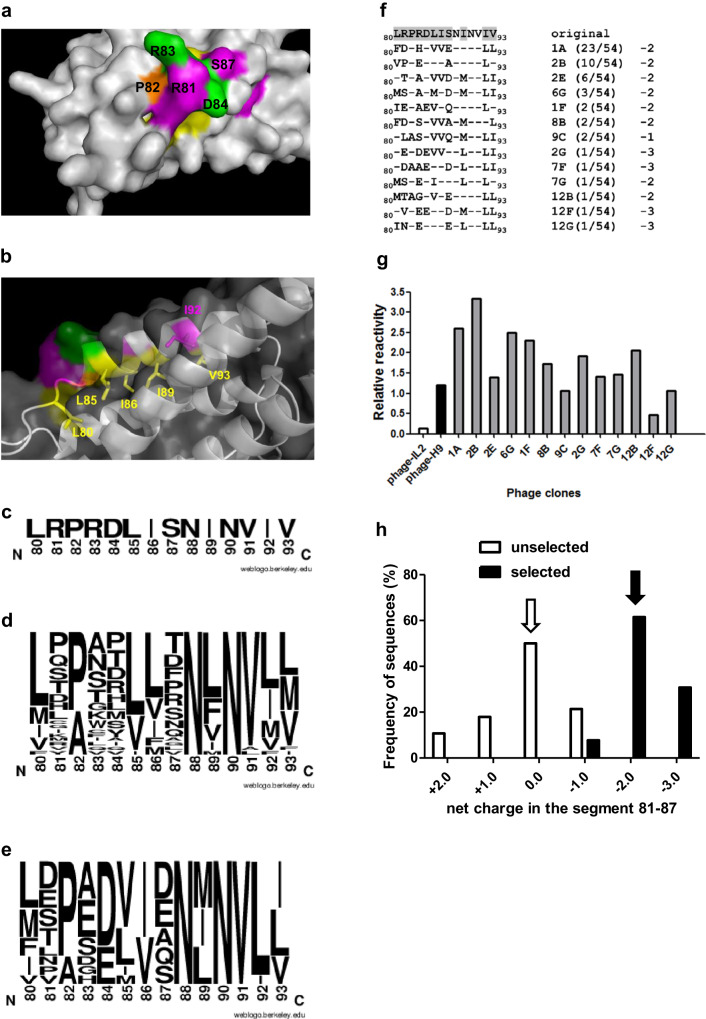
Phage selection from the secondary library S2. Library design is illustrated in **a**, **b**. The human IL-2 structure (from PDB code 2B5I) is represented in grey. The figures were generated with Pymol. Mutational hotspots identified in the initial screening (R81, S87, I92) are highlighted in magenta. Neighbour protruding residues (R83, D84) are shown in green. Adjacent non-exposed residues appear in orange (P82) and yellow (L80, L85, I86, I89, V93). Positions 81, 83, 84 and 87 were totally randomized in S2 library and P82 was replaced by the mixture Pro/Ala (**a**). L80, L85, I86, I89, I92 and V93 were substituted by a mixture of hydrophobic residues (Ile, Leu, Met, Phe and Val). Their side chains are represented as sticks under a semi-transparent surface (**b**). The library was constructed by Kunkel mutagenesis on a hIL-2 gene-containing phagemid template. Phages were panned on the immobilized extracellular domain (ECD) of IL-2R beta chain (three rounds). The original sequence of hIL-2 segments 80–93 is shown in (**c**). The web logos represent unselected library diversity (**d**) and the global sequence profile of selected clones (**e**), with the size of each letter being proportional to the frequency of the corresponding amino acid at a given position. Thirteen different sequences were retrieved after selection (**f**). The original sequence (80–93) appears in the first line, with targeted residues shaded in grey. Selected sequences are shown below. Short lines indicate the conservation of the original residue. The numbers between parentheses represent the frequency of each sequence within the sample of 54 selected clones. Net charge in the segment 81–87 of each sequence is also shown. The reactivity of purified phage preparations from selected clones was evaluated by ELISA on polyvinyl chloride microtitration plates coated with either beta chain ECD or anti-*c-myc* tag 9E10 antibody. Bound phages were detected with an anti-M13 antibody conjugated to horseradish peroxidase (**g**). Relative reactivity was calculated as the ratio between signals obtained with the beta chain and with 9E10. Phage-displayed hIL-2 and H9 superkine were used as controls. There was a shift towards higher negative charges in segment 81–87 upon selection (**h**). White and black arrows indicate the most frequent net charge before and after selection, respectively.

Secondary library S2 resembled S1 diversity at positions 81–84 and 87 (Fig. 3a), but incorporated variability at the inner hydrophobic layer adjacent to the beta chain interface, which included the third identified hotspot (I92, only partially solvent-exposed) and had been previously postulated to influence binding^10^ (Fig. 3b). L80, L85, I86, I89, I92 and V93 were replaced by a mixture of hydrophobic residues (F/I/L/M/V) that could cause a protein core rearrangement. S2 library size was 2.6 × 10^9^, slightly lower than the theoretical combinatorial diversity of the targeted region (5 × 10^9^). Sequencing analysis of 29 cytokine-displaying library clones revealed the presence of one wt hIL-2 sequence (Fig. 3c) and 28 unique mutated variants, having the expected diversity (Fig. 3d). The presence of only three fortuitous changes (V91L/V91A in two clones and a replacement outside the targeted region in other) showed library correctness.

The global sequence diversity in the targeted segment of clones obtained after phage selection from S2 library on beta chain ECD revealed again common features (Fig. 3e). Negatively charged aa at positions 81, 83, and 87 were abundant, as in S1 library selected clones. Residue 84 was always Glu or Asp. Diverse hydrophobic residues eventually replaced L80, L85, I86, I89, and V93. The change I92L was uniformly found in all but one the selected variants. There were 13 unique mutated variants among 54 analysed clones (Fig. 3f). The predominant one was found 23 times and other sequences were also repeated. All selected variants showed increased beta chain binding compared to wt phage-displayed hIL-2. Several were more reactive than phage-displayed H9 superkine (Fig. 3g). A remarkable charge shift in the segment 81–87 was observed again among the selected variants, all negative (mostly −2 and −3) (Fig. 3h).

### Selected IL-2 variants combined increased beta chain binding ability with low aggregation propensity, high expression levels and stability

Predominance of a sequence (or sequence motif) among selected clones is related to target binding capacity, but also to other factors modulating gene expression (codon usage, mRNA secondary structures) and protein display (intrinsic protein stability, folding speed, propensity to misfolding/aggregation/degradation), and even to fortuitous over-representation of some variants in the library. This fact, together with the difficulties to control the precise quantity and quality of phage-displayed molecules, made it necessary the use of alternative formats to study the binding properties and biological activity of the selected variants.

**Fig. 4:**
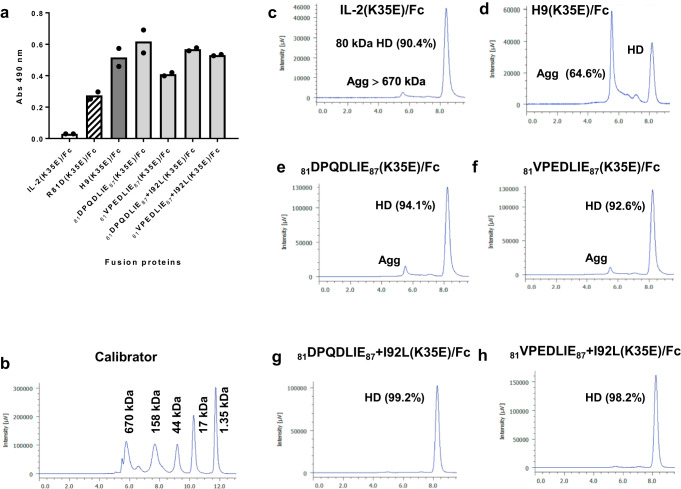
Characterization of recombinant proteins derived from S1 library screening. Recombinant proteins comprising hIL-2 variants designed upon S1 library screening, fused to a human IgG1 Fc region, were produced by transient transfection of HEK-293T cells adapted to grow in suspension, and purified by protein A affinity chromatography. Control fusion proteins derived from hIL-2, from its single-mutated variant (R81D) and from the H9 superkine, were included. The reactivity of the purified proteins (31.25 ng/mL) was evaluated by ELISA on polyvinyl chloride microtitration plates coated with a recombinant fusion protein comprising human IL-2R beta subunit extracellular domain and mouse IgG2a Fc (**a**). Bound proteins were detected with an anti-human IgG antibody conjugated to horseradish peroxidase. Bars indicate the mean absorbance values of two replicates, while dots represent individual data points. The aggregation status of the proteins was determined by size exclusion chromatography in a TSKgelG3000SWXL column. The percentages of major peaks corresponding to homodimers (HD) or large aggregates (Agg) are shown. A calibrator comprising five molecules of known molecular weight (Thyroglobulin 670 kDa, IgG 158 kDa, Ovalbumin 44 kDa, Myoglobin 17 kDa, B12 1.35 kDa) was analyzed in parallel (**b**). Elution profiles of the recombinant fusion proteins are shown in (**c**–**h**).

Phage-derived beta super-binders were characterized as bivalent cytokine-Fc (human IgG1) fusion proteins, expressed by transient transfection of HEK-293T cells adapted to grow in suspension. The replacement K35E (known to improve secretion and folding of IL-2-derived recombinant proteins produced by mammalian cells)^14^ was introduced in all of them. The first two aa of hIL-2 (Ala-Pro), absent in phage-displayed molecules, were restored in these constructs. The first wave of fusion proteins, designed on the basis of S1 library selection output, included frequently selected mutations (R81D/V, R83Q/E and S87E), and had a net negative charge (−3) in the segment 81–87. These variants were named _81_DPQDLIE_87_(K35E)/Fc and _81_VPEDLIE_87_(K35E)/Fc, reflecting their primary sequences. The introduction of the replacement I92L, retrieved from the initial soft-randomization library, gave rise to recombinant proteins _81_DPQDLIE_87_ + I92L(K35E)/Fc and _81_VPEDLIE_87_ + I92L(K35E)/Fc. The four fusion proteins showed increased beta chain binding in enzyme-linked immunosorbent assay (ELISA) as compared to the fusion protein containing hIL-2 (subsequently identified as IL-2(K35E)/Fc), at levels similar to the control H9(K35E)/Fc fusion protein based on H9 superkine (Fig. 4a).

The additional control protein R81D(K35E)/Fc, containing only one of the H9 mutations (R81D), showed a moderately increased beta chain reactivity compared to IL-2(K35E)/Fc, but below that of H9(K35E)/Fc (Fig. 4a). This finding indicated a functional contribution of R81D (a charge reversal mutation) to H9 binding properties. CD122 reactivity of fusion proteins comprising phage-derived super-binders was higher than the one of R81D(K35E)/Fc, suggesting that the accumulation of negative charges arising from the selection is more effective than R81D alone.

There were differences between the developability profiles of the fusion proteins. Yields of purified proteins containing the phage-derived beta super-binders were in the same range as the one of IL-2(K35E)/Fc (70–120 mg/L). H9(K35E)/Fc yield was consistently below 30 mg/L, suggesting an intrinsic manufacturability disadvantage of H9 superkine. Differences in the aggregation status of fusion proteins, shown by size exclusion chromatography (SEC) using a calibrator (Fig. 4b) as a reference, were remarkable. While IL2(K35E)/Fc consisted of 90.4% of homodimers formed through Fc-self-dimerization (Fig. 4c) and a minor peak of high molecular weight aggregates, H9(K35E)/Fc was heavily aggregated (Fig. 4d). Proteins _81_DPQDLIE_87_(K35E)/Fc and _81_VPEDLIE_87_(K35E)/Fc were predominantly found as homodimers (92–95%) (Fig. 4e, f). The change I92L conferred further resistance to aggregation, with homodimers representing more than 98% of _81_DPQDLIE_87_ + I92L(K35E)/Fc and _81_VPEDLIE_87_ + I92L(K35E)/Fc (Fig. 4g, h). The strong aggregation propensity of H9(K35E)/Fc, unique among the whole set of proteins, was confirmed by SDS/PAGE under non-reducing conditions. In such an experimental setting, this was the only protein keeping remnant denaturation-resistant aggregates (Supplementary Fig. 1a), absent after exhaustive denaturation upon reducing treatment (Supplementary Fig. 1b).

As the fusion proteins containing replacements selected from S1 library were similar to H9(K35E)/Fc in terms of beta chain binding ability (Fig. 4a), the possibility of further improvements was explored. A second wave of fusion proteins was designed on the bases of S2 library output. Two of them included hIL-2 sequences from phage-displayed variants 1 A and 2B (Fig. 3f). Both recurrently selected molecules exhibited the highest CD122 reactivities and a negative net charge of −2 in the segment 81–87. Interestingly, 2B did not share any mutation with H9, representing a different structural solution for CD122 binding increase. This variant was further optimized by incorporating S87E, frequently selected from S1/S2 libraries, which results in a higher net negative charge (−3) in its 81–87 segment. The fusion proteins were:

_80_FDPHDVVE_87_ + I92L + V93L(K35E)/Fc

_80_VPPEDLIA_87_ + I92L(K35E)/Fc

_80_VPPEDLIE_87_ + I92L(K35E)/Fc

As several selected sequences (including 1A) overlapped with H9 superkine, the combination of H9 mutations with replacements emerging from phage screening was also studied. L80F, R81D, L85V and I86V (changes shared between H9 and at least some selected phage clones) were included. R83 was replaced by either Ala or Glu (frequently selected), and the recurrent change S87E was also included. Since residue 92 was Phe in H9, but L92 predominated upon phage selection, both Phe and Leu were tested. The set of designed variants was:

_80_FDPADVVE_87_ + I92F(K35E)/Fc (net charge of −3 in the segment 81–87)

_80_FDPADVVE_87_ + I92L(K35E)/Fc (−3)

_80_FDPEDVVE_87_ + I92F(K35E)/Fc (−4)

_80_FDPEDVVE_87_ + I92L(K35E)/Fc (−4)

**Fig. 5:**
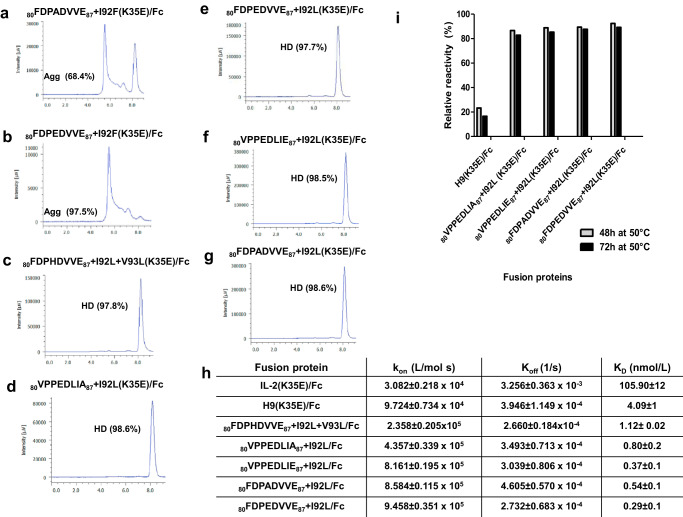
Characterization of recombinant proteins derived from S2 library screening. Recombinant proteins comprising hIL-2 variants designed upon S2 library screening, fused to a human IgG1 Fc region, were produced by transient transfection of HEK-293T cells adapted to grow in suspension, and purified by protein A affinity chromatography. The aggregation status of the proteins was determined by size exclusion chromatography in a TSKgelG3000SWXL column (**a**–**g**). The percentages of major peaks corresponding to homodimers (HD) or large aggregates (Agg) are shown. Association and dissociation kinetic constants, as well as dissociation equilibrium constants, were measured using a Biacore T200 instrument and a biosensor chip coated with a recombinant fusion protein comprising IL-2R beta subunit extracellular domain (ECD) and mouse IgG2a Fc (**h**). The stability of the fusion proteins under thermal stress was assessed by ELISA after incubating them at 50 °C (**i**). Purified proteins (15.6 ng/mL) were incubated on polyvinyl chloride microtitration plates coated with the recombinant fusion protein IL-2R beta ECD/mouse IgG2a Fc. Bound proteins were detected with an anti-human IgG antibody conjugated to horseradish peroxidase. The fusion protein derived from H9 superkine was included as a control. Untreated proteins were also evaluated and used as references to calculate the relative reactivity (%) of each heated sample.

This second set of fusion proteins was heterogeneous in terms of expression levels and aggregation propensity. Proteins including F92 were poorly expressed (below 30 mg/L) and heavily aggregated (Fig. 5a, b). Therefore, they were excluded from further analysis. The remaining fusion proteins, all having L92, were produced as expected (85–105 mg/mL) and had a very low aggregation propensity (>97% of homodimer, Fig. 5c–g). SDS/PAGE analysis revealed the presence of denaturation-resistant aggregates in all aggregation-prone fusion proteins containing I92F (Supplementary Fig. 2). While the binding ability of the fusion protein _80_FDPHDVVE_87_ + I92L + V93L(K35E)/Fc (derived from phage clone 1A) was rather similar to the one of the H9(K35E)/Fc fusion protein, all the remaining S2 library-derived fusion proteins exhibited clearly higher CD122 reactivity in ELISA than both IL2(K35E)/Fc and H9(K35)/Fc (Supplementary Fig. 3).

Real-time analysis of binding kinetics of the fusion proteins to beta chain ECD, fused to a mouse IgG2a Fc and immobilized on a Biacore sensor chip, showed that dissociation equilibrium constants of fusion proteins derived from our beta super-binders decreased by around 100-fold or even more (down to subnanomolar range) as compared to IL-2(K35E)/Fc (Fig. 5h). Such decreases are higher than the one exhibited by H9(K35E)/Fc (approximately 25-fold). The difference was mainly attributed to faster association kinetics. Whereas k_off_ values of all molecules derived from beta super-binders (including H9) were in a narrow range around 3 × 10^−4^ 1/s (one order below the hIL-2 fusion protein), k_on_ values of fusion proteins including phage-derived beta super-binders were 2–10-fold higher than the one of their H9-containing counterpart, and 7-31-fold above IL-2(K35E)/Fc.

An additional advantage of the currently developed set of proteins was their improved stability. Fusion proteins derived from the four highest affinity beta super-binders kept CD122 reactivity by more than 80% after being incubated up to 72 h at 50 °C, whereas H9(K35E)/Fc lost more than 70% of its binding ability upon treatment (Fig. 5i).

The sharp contrasts in the developability profiles of IL-2 derived variants were confirmed when fused to the human IgG1 Fc(LALA) domain, which includes mutations L234A and L235A to abrogate Fc-mediated effector functions, and produced through stable lentiviral transduction of HEK-293 cells. After picking the best clones, those producing fusion proteins based on our beta super-binders were shown to secrete 80–150 mg/L of them, in the same range as the ones producing the IL-2-based protein (Supplementary Fig. 4). The variant _80_FDPEDVVE_87_ + I92L(K35E)/Fc(LALA) was produced at even higher levels (145–185 mg/L). The fusion protein containing H9 was secreted at significantly lower levels than the rest (20–27 mg/L).

### Position 92 is critical for the improved manufacturability properties of IL-2-derived beta super-binders

**Fig. 6:**
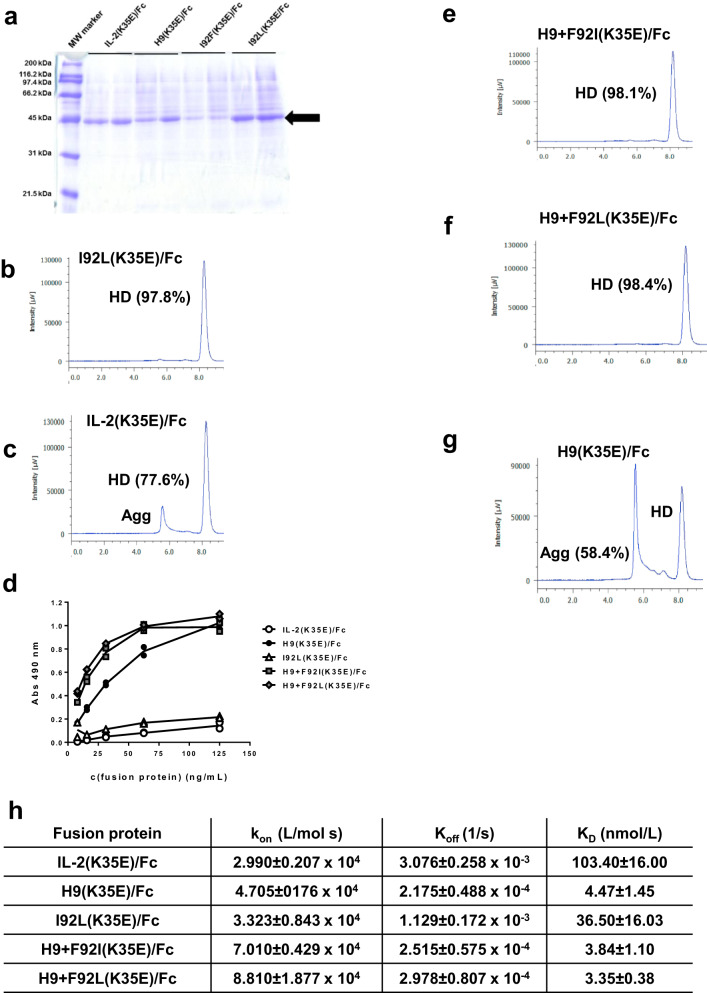
Characterization of recombinant proteins derived from hIL-2 variants with mutations at position 92. A panel of recombinant proteins comprising hIL-2 and H9 superkine with or without additional replacements at position 92, fused to a human IgG1 Fc region, was produced by transient transfection of HEK-293T cells adapted to grow in suspension. Two independent transfection experiments were performed for each variant. Supernatants were collected seven days post-transfection and analysed by SDS/PAGE in a 12% gel under reducing conditions (**a**). 40 µL of treated supernatant was applied to each lane. The molecular mass of each protein in the molecular weight marker is shown on the left side. The arrow indicates the position of the bands corresponding to recombinant fusion proteins. The recombinant proteins were purified by protein A affinity chromatography. The aggregation status of the proteins was determined by size exclusion chromatography in a TSKgelG3000SWXL column (**b**, **c**, **e**–**g**). The percentages of major peaks corresponding to homodimers (HD) or large aggregates (Agg) are shown. The reactivity of purified proteins was titrated by ELISA on polyvinyl chloride microplates coated with a recombinant fusion protein (IL-2R beta/mFc) comprising IL-2R beta subunit extracellular domain and mouse IgG2a Fc (**d**). Bound proteins were detected with an anti-human IgG antibody conjugated to horseradish peroxidase. Symbols represent the absorbance values of two replicates of each protein concentration. Association and dissociation kinetic constants, as well as dissociation equilibrium constants, were measured using a Biacore T200 instrument and a biosensor chip coated with IL-2R beta/mFc (**h**).

The above described results pointed to a beneficial effect of L92 for developability, and a deleterious impact of F92. Both observations arose from proteins with several replacements in the neighbourhood of residue 92. The study of single-mutated variants (I92L/I92F) revealed their direct effects. The yield of the fusion protein containing I92L-hIL-2 was high (143 mg/L of purified protein in transient transfection), while its I92F-containing counterpart was poorly produced (Fig. 6a) and could not be purified. Characterization of this variant was impossible, but production failure was quite informative by itself, showing the association between F92 and a poor developability profile. I92L-containing fusion protein was shown to have a lower aggregation propensity (more than 97% of homodimer in SEC) (Fig. 6b) than IL-2(K35E)/Fc (Fig. 6c). Its beta chain reactivity was only minimally increased over the wt range (Fig. 6d). Such results suggested that I92L had been selected from libraries due to its positive impact on protein synthesis, folding and/or stability rather than because of a direct influence on binding to the selector molecule.

The effect of I92F could explain the developability problems of the fusion protein based on H9 superkine. This was explored through two additional recombinant versions of H9(K35E)/Fc, where F92 was replaced by either the original Ile or the phage-selected Leu. Both kept the increased beta chain binding (Fig. 6d), but exhibited improved yields (59 and 125 mg/L for F92I and F92L-containing proteins respectively, as compared to less than 30 mg/L for H9(K35E)/Fc). They also showed lower aggregation propensity (Fig. 6e, f) than H9(K35E/Fc) (Fig. 6g). SPR measurements using Biacore^TM^ confirmed that the affinity changes associated to either the replacement I92L in IL-2 or the removal of F92 in the superkine context were minimal (Fig. 6h). Residue F92 was thus not required to obtain a beta super-binder, but was fully responsible for the poor intrinsic developability of H9 superkine. SDS/PAGE analysis under non-reducing conditions confirmed that the typical H9(K35E)/Fc denaturation-resistant aggregates disappeared after replacing F92 with either Ile or Leu (Supplementary Fig. 5).

### In silico exploration of the structural bases of beta chain binding and developability properties of mutated IL-2 variants

**Fig. 7:**
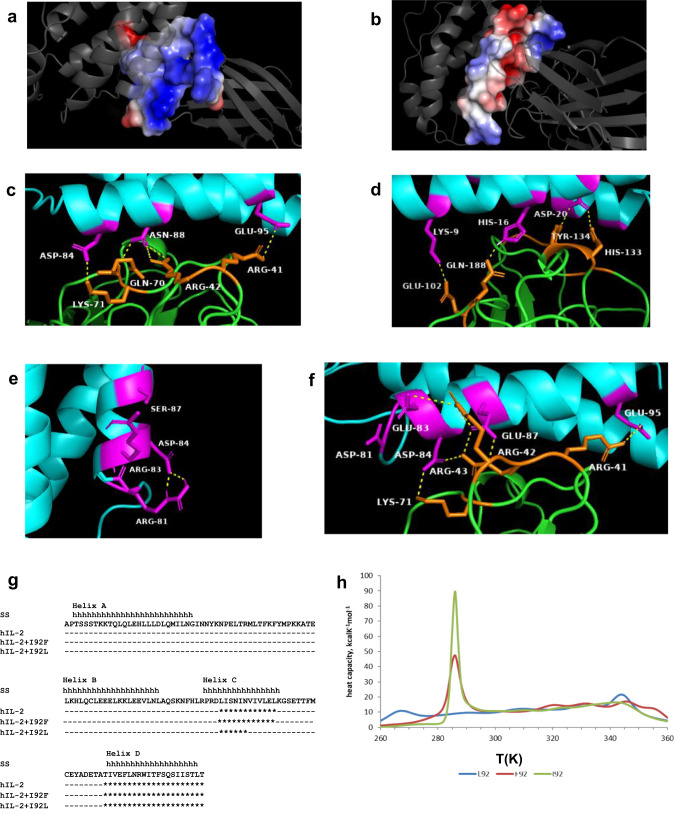
In silico exploration of the structural bases of CD122-driven IL-2 directed evolution. Electrostatic potential at wild-type (wt) IL-2/IL-2R beta chain interface is analysed in a-b. The backbones of both molecules are represented as grey cartoons. The surface of residues belonging to the interface at either the beta chain side (**a**) or the IL-2 side (**b**) is coloured according to its electrostatic potential. Colour intensity reflects the density of positive (blue) and negative (red) charges. Panels **c** and **d** represent two different views of the original interface. IL-2 and IL-2R beta chain backbone cartoons are shown in cyan and green respectively. Side chains of the residues directly involved in the interaction between them are represented as magenta (IL-2) and orange (beta chain) sticks. Yellow dotted lines indicate polar contacts between them. Residues engaged in these contacts are labelled. The structure of free IL-2, with the side chains of residues randomized in phage libraries shown as sticks and labelled, appears in (**e**). Intra-molecular polar contacts between R81 and D84 are indicated. A model of the reshaped interface between one of the beta super-binders (_80_FDPEDVVE_87_ + I92L) and CD122 is represented in (**f**). The new polar contacts established by the residues E83 and E87 (arising from IL-2 phage selection) with positive residues at the beta chain can be appreciated. The search for aggregation-prone regions (amyloid-forming) by WALTZ method underscored two motifs in helices C and D of hIL-2 and its F92-containing version (indicated by asterisks). The length of the putative aggregation-prone motif within helix C was reduced by 50% upon the introduction of the change I92L (**g**). 3D models of hIL-2 variants with replacements at position 92 (after energy minimization with Moe using the amber force field) were used to perform coarse grained Multiplexed Replica Exchange Molecular Dynamics simulations with the UNRES force field. Heat capacity profiles for each variant were constructed according to these results (**h**).

The analysis of the original hIL-2/CD122 interface revealed a high density of positive charges at the human beta chain side (Fig. 7a), and a mixture of positive and negative patches at the hIL-2 side (Fig. 7b). The low intrinsic affinity of wt hIL-2 for CD122 is presumably associated with the lack of an optimal electrostatic complementarity. Despite this suboptimal context, the interaction network between both molecules includes polar contacts between charged residues (Fig. 7c, d): K9(IL-2)-E102(CD122), D20(IL-2)-H133(CD122), D84(IL-2)-K71(CD122), E95(IL-2)-R41(CD122). Most pairs consist of a negatively charged side chain in IL-2 and a positive residue in CD122. Accumulation of negatively charged aa along the segment 81–87 in beta super-binders, together with counter-selection of R81 and R83, resulted in a globally improved electrostatic complementarity.

One of the negative residues of IL-2 interface (D84) was targeted in phage-displayed libraries. Variants selected on the beta chain tend to keep the original Asp or Glu (also negatively charged), confirming the relevance of its involvement in electrostatic interactions. On the other hand, R81 and R83 (positively charged) were replaced by a plethora of aa, sometimes negatively charged, but also neutral. Besides the impact of these substitutions in creating a more favourable environment at the hIL-2 side of the interface, R81 replacement could have a specific effect. R81 can establish electrostatic interactions with D84 in free hIL-2 (Fig. 7e). Disruption of this bond is required for the formation of the critical D84(IL-2)-K71(CD122) ionic bond, implying an energetic barrier for complex formation. R81 replacement thus favours cytokine-receptor engagement indirectly, by leaving D84 free to interact with CD122. Some of the emerging negatively charged residues can establish additional electrostatic interactions with CD122 (Fig. 7f). This is the case of E83, present in some of the super-binders. More importantly, the recurrent presence of negative residues (Glu/Asp) at position 87 (originally neutral) was shown to result in a network of ionic interactions and hydrogen bonds engaging both R42 and R43 in CD122 (Fig. 7f).

Regarding the critical influence of aa 92 in the developability profile, the first analysis relied on a close inspection of the four amphiphilic helices that compose the wt molecule, since global IL-2 structure and function are known to be strongly dependent on the integrity of its typical alpha-helical secondary structure^16^. While the composition of helices A and B resulted in a high local alpha-helical propensity, calculated by using three different scales, primary sequences of helices C and D were shown to be less favourable for alpha-helix formation (Supplementary Fig. 6a) and exhibited a relatively high propensity to adopt a beta-sheet secondary structure (Supplementary Fig. 6b). The crucial effect of aa 92 was linked to its location within the helix C (P82-K97), which includes five beta-branched hydrophobic residues with low helical propensity (Ile and Val). In such a suboptimal context the introduction of a Leu residue, having a high intrinsic propensity to form alpha-helices^17^, is expected to facilitate the proper folding. This effect would not happen when I92 is substituted by Phe (another poor contributor to alpha-helix formation). Besides its low intrinsic alpha-helical propensity, the analysis of F92 in the context of the whole molecule revealed that the accommodation of this bulky residue results in a severe entropy loss, as only a few of the multiple possible rotamers are compatible with packing within the four alpha-helix-bundle (Supplemantary Fig. 7).

Additional in silico approaches were used to study the relevance of aa 92 for aggregation and thermal stability, respectively. Two aggregation-prone regions were identified in helices C and D of hIL-2 and its F92-containing version by using the WALTZ method^18^, which is based on the search for experimentally determined amyloid-forming hexapeptide sequences within the protein under study. The length of the putative aggregation-prone motif within helix C was reduced by 50% upon the introduction of the change I92L (Fig. 7g). This finding is presumably linked to the above referred local stabilization of the alpha helix, since proper folding and aggregation mediated by transiently exposed aggregation-prone motifs are competing processes. On the other hand, an exploration of modelled structures of IL-2 variants with mutations at position 92, using Coarse Grained Multiplexed Replica Exchange Molecular Dynamics simulations with the UNRES method^19^, revealed a structural transition in the temperature range 280–290 K for the molecule containing I92F (Fig. 7h). Even though a transition in the same temperature range was also observed for the original hIL-2 containing I92, the transition-associated energy- as appreciated from the heat capacity profiles- is approximately half in the case of I92F version, suggesting lower conformational stability of this mutated variant (Fig. 7h). This phenomenon was not reproduced for the mutated variant containing I92L, for which the first major transition occurred at a much higher temperature (344 K) (Fig. 7h). Taken together, the above described computer-assisted findings are fully consistent with the experimental results showing positive and negative impacts of replacements I92L and I92F on both hIL-2 aggregation resistance and stability.

### Phage-derived IL-2 variants are potent in vitro stimulators of cells displaying beta/gamma IL-2 receptor

**Fig. 8:**
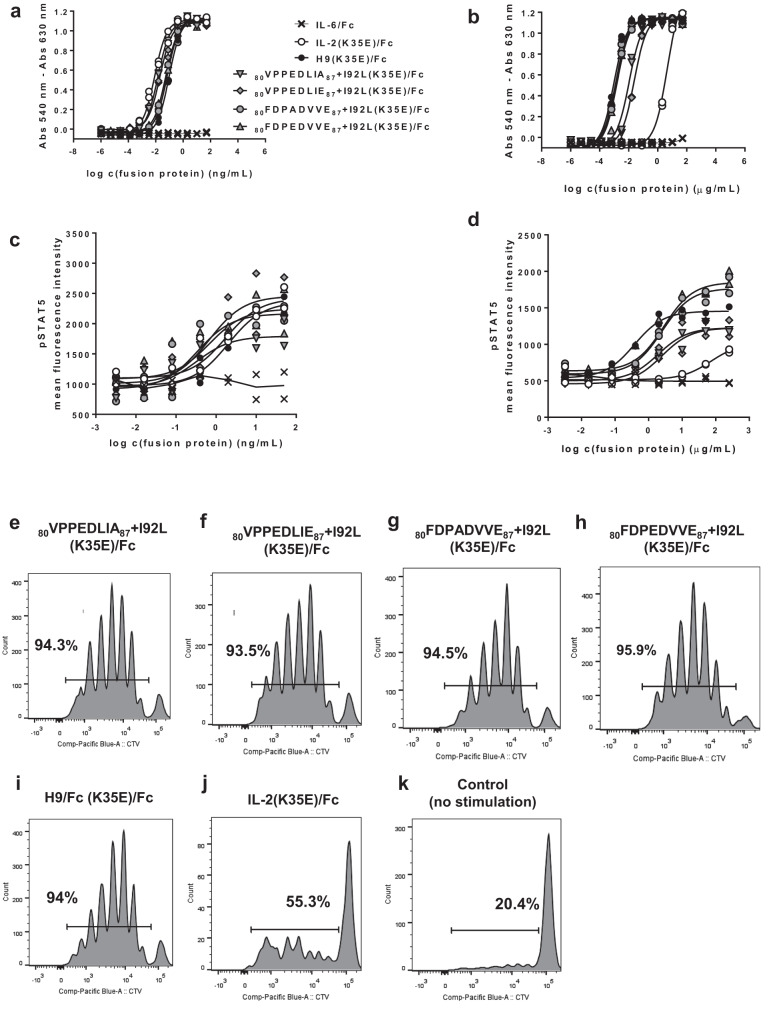
In vitro biological activity of IL-2-derived recombinant fusion proteins. Mouse CTLL-2 cells were grown during 48 h in the presence of serial dilutions of recombinant Fc-fusion proteins comprising our IL-2 beta super-binders. Reference proteins IL-2(K35E)/Fc and H9(K35E)/Fc were also included. Fc-fused IL-6 was used as a negative control. Cells were stained with Alamar Blue dye and the proliferation curves were constructed by plotting the difference between absorbances at 540 and 630 nm as a function of fusion protein concentration (**a**). Two replicates of each protein concentration were analysed. Symbols indicate the values of both determinations. A similar experiment was performed on CTLL-2 cells transduced with lentiviral particles encoding a sgRNA suitable to ablate expression of IL-2R alpha subunit gene (CD25-KO CTLL-2 model) (**b**). Both CTLL-2 cells (**c**) and their CD25-KO counterparts (**d**) were stimulated during 40 min with the set of recombinant proteins, fixed, permeabilized and stained with an APC-labelled antibody against phosphorylated STAT5. The resulting fluorescence signals (determined by flow cytometry) measured the signalling capacity of the proteins on each cell type. Two replicates of each protein concentration were analysed. Symbols indicate the values of both determinations. Purified CD8+ cells from mice lymph nodes were CTV-labeled and stimulated in vitro with recombinant Fc-fusion proteins containing our IL-2-derived beta super-binders (**e**–**h**). Cells treated with similarly formatted H9 superkine (**i**) and hIL-2 (**j**) proteins were included as references. Non-stimulated cells (**k**) were analyzed as negative control (basal proliferation). Fluorescence dilution was determined by flow cytometry.

In vitro assessment of the proliferation-inducing activity on CTLL-2 cell line of mouse origin (the classical experimental system used to characterize IL-2 and related molecules)^20^ revealed that all the tested fusion proteins are active in a dose-dependent manner (Fig. 8a). The resulting proliferation curves overlapped, and concentrations of every protein inducing half-maximal proliferation were in a rather narrow range (8–94 pg/mL). As CTLL-2 cells display the trimeric high affinity IL-2R that includes the alpha chain, they were highly sensitive to IL-2R stimulation and the contribution of increased beta chain binding to the cellular response was not evident. On the other hand, functional differences were readily observed in a similar experiment using CTLL-2 cells genetically modified to ablate alpha chain expression (CD25-KO CTLL-2). In such a scenario, the fusion proteins derived from all beta super-binders, including the control H9(K35E)/Fc were much more potent stimulators of cell proliferation than IL-2(K35E)/Fc (Fig. 8b). While more than 4 μg/mL of the latter were required to induce half-maximal proliferation, fusion proteins based on beta super-binders were similarly effective at lower levels (below 25 ng/mL). The whole set of experiments illustrated how beta chain binding gains functional relevance in the context of cells displaying the intermediate affinity beta/gamma dimeric receptor.

Similar results were obtained when measuring STAT5 phosphorylation as an indicator of cell signalling through IL-2R mediated by the set of fusion proteins. All of them, including the control protein IL-2(K35E)/Fc, induced dose-dependent STAT5 phosphorylation in CTLL-2 cells (Fig. 8c). In the case of CD25-KO CTLL-2 model, treatment with fusion proteins based on beta super-binders induced phosphorylation in a much more efficient way than IL-2(K35E)/Fc (Fig. 8d), reinforcing the idea that the signalling advantage associated to increased beta chain affinity is restricted to cells displaying beta/gamma receptor. While 72 μg/mL of IL-2(K35E)/Fc was estimated as the required dose to reach half-maximal signalling, less than 3 μg/mL of fusion proteins comprising beta super-binders were able to induce equivalent STAT5 phosphorylation levels. The differences between diverse beta super-binders could be related to variations in their relative affinities against mouse IL-2R beta subunit, their net binding capacities to the membrane-anchored assembled receptor, and/or the influence of mutations on internalization/recycling rates in living cells.

Fusion proteins derived from our beta super-binders induced strong proliferation (more than 93%) of purified mouse CD8 + T cells having the dimeric beta/gamma receptor (Fig. 8e–h). Such activity was not distinguishable from the one of H9(K35E)/Fc (Fig. 8i), but clearly outperformed IL-2(K35E)/Fc, which only induced 55.3% of proliferation (Fig. 8j) and low levels of basal proliferation (Fig. 8k). This experiment was a more realistic approach to show in vitro super-agonism directly on the ultimate target cells: CD8 + T lymphocytes isolated from a living organism.

### In vivo experiments revealed highly potent immunomodulatory and anti-tumour properties of beta super-binders

**Fig. 9:**
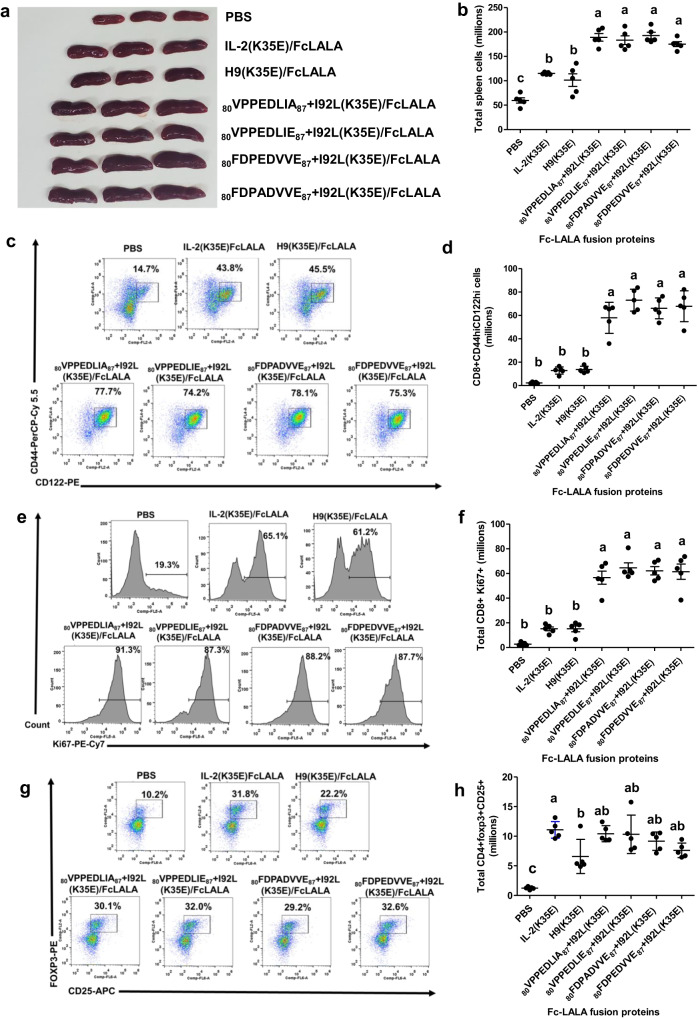
In vivo expansion of immune cell populations induced by IL-2-derived fusion proteins. Four groups of five mice each received daily injections (during four days) of recombinant proteins comprising IL-2-derived beta super-binders fused to human Fc(LALA). Two additional groups were treated with similarly formatted proteins containing either hIL-2 or H9 superkine. Mice from a control group received PBS injections. All the animals were sacrificed at the fifth day, and their spleens were collected and macerated for cell count and characterization by flow cytometry. Spleen enlargement induced by treatment with fusion proteins (three representative spleens from each group) can be appreciated in picture (**a**). The cell content of the whole set of spleens is represented in (**b**). The expansion of the effector T cell population (CD8+CD44hiCD122hi) induced by fusion proteins, was assessed by flow cytometry. Representative dot plots from one animal of each group are shown in (**c**). The numbers indicate percentages of CD44hiCD122hi cells within the CD8+ population. Total CD8+CD44hiCD12hi cells in the whole set of animals are represented in (**d**). The increase in proliferating effector cells (CD8+ Ki67+), assessed by flow cytometry, is shown in (**e**) (one representative histogram per group, with numbers indicating percentages of proliferating cells) and (**f**) (total proliferating effector cells in the whole experimental set). The expansion of the Treg population (CD4+ CD25+ Foxp3+) induced by fusion proteins, also assessed by flow cytometry, is shown in (**g**) (one representative dot plot per group, with numbers indicating percentages of CD25+ Foxp3+ cells within CD4+ population) and (**h**) (total Treg cells in the whole experimental set). Lines represent mean values and error bars indicate SD within each group of five animals receiving the same treatment. One-way ANOVA followed by a Tukey multiple-comparison test (*p* < 0.05) was used for comparisons between groups. Different letters mean significant differences between groups.

The super-agonist capacity of the newly described beta super-binders on mouse cells (see the previous section) allowed testing their in vivo effects in mouse models. The use of Fc(LALA)-fusion proteins ruled out the potential influence of Fc-mediated effector functions on immunological effects^21^, which could be solely attributed to the direct stimulation of immune cells by the IL-2 moieties. Repeated in vivo injections of Fc(LALA)-fusion proteins containing our beta super-binders resulted in strong immunostimulation, indicated by spleen enlargement in treated mice (Fig. 9a), as it has been described for highly immunostimulatory immune complexes^22^. Their effects were higher than the ones mediated by fusion proteins containing hIL-2 and even H9 superkine. Spleen size differences were largely attributed to cell content variations (Fig. 9b). FACS analysis revealed a large expansion of the activated effector CD8 + T cell population (CD44hiCD122hi) upon treatment with Fc(LALA)-fused phage-derived beta super-binders (Supplimentary Fig. 8, Fig. 9c, d). Such expansion was not observed with control proteins containing hIL-2 and H9, and was associated with enhanced proliferation, as shown by the increase in proliferating CD8 + T cells (Ki67 +) (Supplementary Fig. 8, Fig. 9e, f). The potent stimulating effects of our variants on immune effector cells were in sharp contrast with the homogeneous ability of all fusion proteins to induce the expansion of CD4+ Treg population (FoxP3 + CD25 + ) (Supplementary Fig. 9, Fig. 9g, h).

The above described results showed a drastic shift in the balance of in vivo immunomodulatory properties of hIL-2-derived fusion proteins upon the introduction of the mutations conferring increased CD122 binding ability: the molecules became much more potent enhancers of the immune effector functions without changes in their regulatory potential. That shift was not evident for the reference H9(K35E)/Fc(LALA) protein in the current experimental setting, showing an advantage of our beta super-binders as biased immunopotentiators in vivo.

Remarkably, the immunostimulatory potential of the H9-derived recombinant fusion proteins was restored after replacing F92 with either the original Ile or the Leu residue selected from phage libraries (Supplementary Fig. 10). As the major effect of these changes was a drastic reduction in the instability and aggregation propensity of the corresponding proteins with only minor modifications of their binding properties (Fig. 6h), this result pointed to biophysical liabilities as the main cause of the lower in vivo activity of H9-containing proteins.

**Fig. 10:**
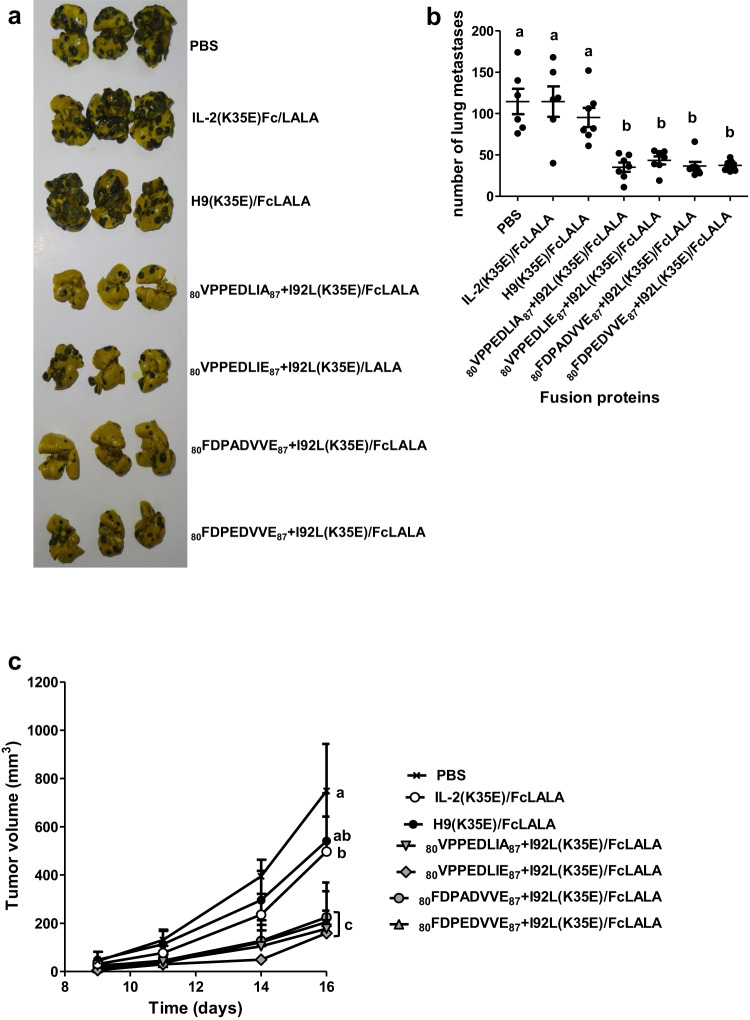
Anti-tumour effects of recombinant fusion proteins containing beta super-binder IL-2 variants. Four groups of seven C57BL/6 mice each were inoculated intravenously with 1 × 10^5^ MB16F0 melanoma cells, and subsequently treated with five daily injections of the recombinant proteins comprising hIL-2-derived beta super-binders fused to human Fc(LALA). Two additional groups were treated with similarly formatted proteins containing either hIL-2 or H9 superkine. Mice from a control group received PBS injections. All the animals were sacrificed at day 21, and their lungs were extracted for counting the number of metastatic nodules. The lungs of three animals of each group are shown in (**a**). The number of metastases of the whole set of experimental animals is represented in (**b**). Two animals, from the groups treated with PBS and IL-2(K35E)Fc(LALA) respectively, died before completion of the experiment and were excluded. Lines represent mean values, and error bars indicate SD within each group. One-way ANOVA followed by a Tukey multiple-comparison test was used for comparisons between groups. Five groups of five BALB/c mice each were inoculated subcutaneously with 1 × 10^5^ CT26 cells in the left flank and subsequently treated with five daily injections of the above described set of recombinant proteins, and with PBS in the case of the control group. After tumours became palpable, their length and width were measured every other day in order to calculate tumor volume until one tumor side reached 18 mm. Tumor volumes are represented in (**c**). Points represent mean values, and error bars indicate SD within each group. For the statistical analysis a two-way ANOVA, followed by a Tukey post-test (*p* < 0.05), was performed. Different letters indicate differences between groups.

The immunomodulatory properties of the fusion proteins were translated into potent in vivo anti-tumour effects in an experimental metastases model. Treatment of mice with repeated injections of the recombinant fusion proteins containing phage-derived beta super-binders prevented the formation of lung metastases after intravenous inoculation of MB16F0 cells (Fig. 10a, b). This effect was not observed for the fusion proteins based on either hIL-2 or H9, although the latter showed a moderate tendency to diminish the number of metastases. Subcutaneous growth of tumours (CT26 colon carcinoma model) was also inhibited by our set of fusion proteins, which were more effective than the control proteins containing IL-2 and H9 (Fig. 10c). No gross signs of toxicity, like weight loss, were detected in any group of treated animals.

## Discussion

The use of H9, and H9-derived proteins, in experimental anti-tumour therapy provided solid proof of the concept of dimeric IL-2R super-agonism resulting in strong effector-biased immunological effects^10,23,24^ Combination of H9 mutations with replacements modulating the interaction with other IL-2R subunits gave rise to partial agonists, potent antagonists and selective agonists^25–27^. We showed that the change I92F, present in all of them, causes diminished stability and high aggregation propensity. However, H9-related proteins have been produced and are biologically active. The extent to which intrinsic biophysical liabilities compromise protein manufacturability depends upon recombinant protein design, stabilizing/destabilizing mutations, fusion partners, host cells, expression conditions and downstream processing (purification/formulation). The current work differs from previous experiences in the use of an Fc-fusion format and mammalian cell expression. Although Fc-fusion has been used to increase hIL-2 in vivo half-life and biological effects^28^, hIL-2/Fc proteins have shown aggregation propensity and exquisite sensitivity to mutations modulating secretion and folding^14^. H9-related proteins had been produced in insect High Five host cells up to now^10,23–26^, and similar expression systems have shown advantages in the case of aggregation-prone difficult-to-express-proteins^29^.

Eliminating the liabilities of therapeutic candidates represents a step forward in the complex way of product development. In the case of IL-2, H9 and related proteins, the replacement K35E improved expression levels and aggregation status^14^. That is why it was included in all the molecules during the current study. This change does not totally preclude aggregate formation, particularly in those proteins with stronger aggregation propensity like H9/Fc. Removal of residue F92 was enough to obtain high expression of a homogeneous protein product. Although this was achieved by reversing the change I92F back to the original Ile, the introduction of L92 was more favourable. I92L likely improves the biosynthesis of IL-2-derived recombinant proteins in mammalian cells, both in quantity and quality, in a similar way to K35E, also identified from phage display screening^14^. None of these replacements emerged from yeast display^10,30^. Such a difference could be due to diverse library construction strategies and sizes but could reflect a higher sensitivity of phage-based systems to biophysical liabilities of displayed proteins.

The selectivity of phage panning for I92L was almost absolute, as many clones selected from the soft-randomization library and the vast majority of those obtained from the secondary library S2 have L92. None contained either F92, previously selected with yeast display and causing expression/aggregation problems, or any other change at this position. Presumably, proteins able to follow rapidly a proper folding pathway and reach a stable conformation have a display advantage. Misfolding/aggregation tend to exclude them from the nascent phage particles. Although selectivity for biosynthesis-improving changes could be a general attribute of phage display, a specific contribution of the quick co-translational secretion system we are using for cytokine display^31^, based on *DsbA* signal peptide^32^, cannot be ruled out.

The replacement I92F emerged from cell sorting of yeast-displayed variants showing stronger CD122 binding, and F92 was postulated to act as a molecular wedge between helix C and helix A, which stabilizes a receptor binding-prone cytokine conformation^10^. Our results do not support such a functional role. The practical implication of our findings is that the molecular determinants for increased CD122 binding can be segregated from biophysical liabilities, allowing the remodelling of H9 and related superkines into molecules with improved developability. Leu at position 92 also has a negligible contribution to binding, and its phage selection is likely to be the sole result of the biosynthesis/secretion/folding improvement. The other mutations in the superkine hydrophobic core selected through yeast display (L80F, L85V, I86V)^10^ also emerged recurrently from phage libraries. Although these changes are not strictly required for increased binding, as several super-binders lack them, they are likely contributors to binding improvement when present.

Besides stringent selectivity at position 92 and hydrophobic core remodelling, CD122-driven pressure on hIL-2 resulted in a global electrostatic change at the interface. Selection output was not limited to a few particular combinations of mutations but included diverse molecules, whose main shared feature was the net negative charge (−2 and higher) in the segment 81–87. S1 library screening, without modifying the hydrophobic core, showed that a better electrostatic complementarity is enough to increase CD122 binding. The first mutation of this kind, R81D (also selected from phage libraries), had been described in H9-family superkines^10^. Even though the hydrophobic core rearrangement was postulated to play a major role in the conformational switch behind superkine evolution^10^, we proved that R81D contributes to the increased binding. The proposed underlying mechanism is the elimination of the energetic barrier that R81-D84 interaction represents for the engagement of D84 by CD122. Clusters of negative residues were even more effective in increasing binding that this single mutation. The existence of multiple structural solutions for improved beta chain binding highlights the plasticity of the interaction interface and the possibility of designing other combinations of mutations based on the general principles described here.

The new beta super-binders showed a higher binding affinity to CD122 (up to one order of magnitude) than H9 superkine in our experimental setting, mainly due to faster association kinetics. Characterization of all molecules in Fc-fusion format and on the same immobilized ligand allowed a fair comparison between them. Differences in the absolute values with the ones reported previously for IL-2 and H9^10^ can be explained by variations in the analyte/ligand used, and the in the experimental conditions. Diverse values have indeed been reported^10,27,33,34^. It can be hypothesized that the faster association of the super-binders results from their better electrostatic complementarity with the beta chain surface. But this output could be influenced by misfolding/aggregation of the H9-based fusion protein, as k_on_ measurement depends on the analyte concentration, and inactive and/or sterically hindered molecules are not available for binding. Therefore, it is difficult to completely separate biophysical liabilities from binding properties. The net differences could result from a combination of diverse factors.

Some of the phage-derived beta super-binders were clearly superior to H9 superkine (as Fc-fusions) in terms of in vivo enhancement of the effector responses and anti-tumour activity, despite in vitro similar stimulatory capacity. Even though such output can be influenced by the way in which their binding properties affect the competition between cell populations within a living organism, the lack of biophysical liabilities seems to be crucial for this advantage. This idea was strongly supported by the rescue of the immunomodulatory capacity of the H9-derived fusion proteins upon the introduction of changes at position 92 able to correct its intrinsic propensity to instability and aggregation. Improved protein stability and solubility might thus have a determinant effect on bioavailability and subsequently on biological activity.

Only a few of the newly identified molecules have been characterized up to now. The discovery of multiple beta super-binders differing in primary sequence, and probably in kinetic parameters and subtle details of their interactions, will allow testing their potential to fine-tune the balance between immune regulation, activation and exhaustion of effector cells (T and NK), and memory generation. Intra-molecular combination of the mutations described here with other sequence and format changes, as well as combination therapy with additional agents, should also be studied. Although no gross signs of toxicity were observed in animal model experiments, a deep toxicology study has to be performed before exploiting the therapeutic potential of the molecules.

To our knowledge, there were no previous examples of the application of phage display to evolve IL-2 variants with biased immunological activity, a field dominated up to now by yeast display and rational design^9,10,12,13,30^. Our results support the notion of phage display being an optimal platform for the directed evolution of engineered cytokines, which combine tailor-made biological activity with an intrinsically high developability potential. Reshaping the chemistry of IL-2 interface with the IL-2R beta chain served both purposes and provided a panel of IL-2R super-agonists able to enhance immune effector functions and anti-tumour activity. Their future as immunotherapeutic drugs remains to be explored. Extending the current experience to diverse phagekines^31^ could provide lead candidates for the development of other immune modulators.

## Methods

### Library construction

Single-stranded DNA corresponding to pCSM+ phagemid containing wt hIL-2 gene (Supplementary Fig. 11)^31^ was obtained from transformed CJ236 *E.coli* cells (*dut*^*−*^ *ung*^*−*^ *thi-*1 *relA1 spoT1 mcrA*/pCJ105 (F’ *cam*^r^)) as described^31^. Kunkel mutagenesis^15^ reactions on 20 µg of this template with antisense mutagenic oligonucleotides(CIGB, Cuba) were performed according to established procedures^35^, and TG1 *E.coli* strain cells (K12_(*lac-pro*), *sup*E, *thi*, *hsd*D5/F’ *tra*D36, *pro*A^+^B^+^, *lac*I^q^, *lac*Z_M15) were electroporated with mutagenesis products to construct the libraries. The sequences of the spiked mutagenic oligonucleotides used for soft-randomization library construction are shown in Supplementary Fig. 12, while degenerate mutagenic oligonucleotides used for full randomization and controlled diversification at selected positions of secondary libraries appear in Supplementary Fig. 13. Spiked oligonucleotides contained 90% of the original nucleotide and 10% of the three other nucleotides at each targeted position, in order to achieve soft-randomization of the protein sequence. Degenerate mutagenic oligonucleotides introduced NNK triplets (coding for a mixture of the 20 aa) at positions to be randomized. Pro/Ala mixture was coded by the degenerate triplet SCG, while mixtures of hydrophobic aa (Phe/Ile/Met/Leu/Val) were coded by NTK triplets.

### Phage selection

Phages displaying IL-2 variants from each library were rescued with M13KO7 helper phage at a 300 mL scale and purified through precipitation with polyethylene glycol, as described^36^. Polystyrene immunotubes (Nunc, Denmark) were coated overnight at 4 °C with 10 µg/mL of human IL-2R beta chain ECD (R&D Systems, USA) in phosphate-buffered saline (PBS). Tubes were blocked with PBS containing 4% (w/v) skim milk powder (M-PBS) during 1 h at room temperature (RT). Purified phages (10^12^ viral particles) were blocked by diluting 1:2 with M-PBS and incubating 1 h at RT. Blocked phages were incubated on blocked immunotubes during 1 h at RT. After washing the tubes 20 times with 0.1% Tween 20 in PBS (PBS-T) and twice with PBS, bound phages were eluted with 100 mmol/L triethylamine during 15 min, and immediately neutralized with Tris 1 mol/L, pH 7.5. Neutralized eluate was used to infect exponentially growing TG1 *E.coli* cells, and selected phages were rescued with M13O7 helper phage at a 50 mL scale^36^. Purified phages were the starting material for the next selection round in the same conditions. The procedure was repeated until the third selection round.

### Phage screening

Non-selected library phages and phages obtained at the end of the selection procedure were used to infect TG1 *E.coli* cells. Phages produced by individual colonies were rescued with M13KO7 helper phage at a 96-well scale^37^. Supernatants were tested by ELISA on polyvinyl chloride microtitration plates coated with 9E10 mAb recognizing the *c-myc* tag fused to displayed proteins in our system, in order to identify cytokine-displaying clones^31^. Variants having stop codons or other changes incompatible with secretion/display, which cannot be present in the library at the protein level, were thus excluded from further analysis. Phage-containing supernatants from cytokine-displaying clones were used to infect XL-1Blue *E.coli* cells (*rec*A1 *end*A1 *gyr*A96 *thi*-1 *hsd*R17 *sup*E44 *rel*A1 *lac* F´ *pro*AB l*ac*IqZ_M15 Tn10 Tet^r^). The resulting colonies were grown to purify phagemids with the minipreps kit (Qiagen, Germany) according to the manufacturer’s instructions. The inserted cytokine-coding genes were sequenced by Microsynth-Seqlab (Germany), and protein sequences were deduced. TG1 *E.coli* cells were transformed with phagemids containing unique sequences of interest and used to rescue phages at a 50 mL scale^31^. Purified phages from individual clones, as well as phage pools obtained before and after selection rounds, were tested by ELISA on microtitration plates coated with either human beta chain ECD (R&D Systems, USA), 9E10 mAb or the irrelevant protein bovine serum albumin (BSA), as described^31^. Relative reactivities, calculated as the ratio between signals obtained with beta chain and with 9E10, were useful to normalize receptor binding ability taking into account display levels of each phage preparation.

### Expression of mutated IL-2 variants as Fc-fusion proteins in HEK-293T host cells adapted to grow in suspension

Mutations in the targeted segments of hIL-2 were introduced by site-directed mutagenesis on the template plasmid pCMX containing wt hIL-2 gene, using suitable mutagenic oligonucleotides (CIGB, Cuba). Mutations and correctness of the rest of the gene were confirmed by DNA sequencing (Microsynth-Seqlab, Germany). Modified IL-2 genes were re-cloned into pCSE-2.6 hIgG1 Fc^38^ through BssHII/NotI restriction sites, and sequenced again. HEK-293T cells adapted to grow in suspension in Freestyle F-17 medium (ThermoFisher, USA) were transiently transfected with the resulting genetic constructs, using linear polyethyleneimine (PEI) as the transfecting agent according to established procedures^38^. Fusion proteins were purified from cell culture supernatants by protein A affinity chromatography.

### Expression of mutated IL-2 variants as Fc(LALA)-fusion proteins by stably transduced HEK-293 cells

IL-2 or mutated IL-2 genes obtained by site-directed mutagenesis (see the previous section) were re-cloned into the pCSE-2.6 hIgG1 Fc(LALA), containing a modified human IgG1 Fc gene coding for an Fc fragment with the replacements L234A and L235A. The whole expression cassette of these vectors, including CMV promoter and the genes coding for a mouse IgG heavy chain signal peptide, mutated or wt IL-2, and human Fc(LALA), was amplified by PCR and cloned into the lentiviral vector pL6WBlast (CIGB, Cuba) through XhoI and EcoRV restriction sites. The sequence of the whole inserted DNA fragment was confirmed by Microsynth-Seqlab, Germany. Adherent HEK-293T cells were co-transfected with each of these genetic constructs and pLPI, pLPII and pLP/VSV-G auxiliary plasmids (Invitrogen, USA), in order to assemble lentiviral particles, which were purified from supernatant by precipitation with polyethylene glycol. The concentration of viral particles was determined by ELISA with DAVIH Ag p24 ELISA kit (LISIDA, Cuba). HEK-293 cells were grown in individual wells of 96-well plates with DMEMF12 medium (Life Technologies, USA) supplemented with 5% (v/v) heat-inactivated fetal bovine serum (FBS), and infected three times every 24 h with lentiviral particles at a multiplicity of infection of 800. Transduced cells from each well were diluted and expanded to five 96-well plates in the presence of blasticidin (2 µg/mL) as the selection drug. Secreted fusion proteins in the supernatants were detected by ELISA on polyvinyl chloride microtitration plates coated with the anti-IL-2 mAb CBIL2.2 (CIGB Sancti Spiritus, Cuba). Bound fusion proteins were recognized by an anti-human IgG antibody conjugated to horseradish peroxidase. Oligoclonal cell populations secreting the highest levels of each fusion protein were cloned by limiting dilution, and clones were tested by ELISA as previously described. The best producer clones were expanded, adapted to grow in suspension in the proprietary medium MB06 (serum-free), and used to produce the fusion proteins, which were purified through protein A affinity chromatography.

### Characterization of binding of recombinant fusion proteins to beta subunit by ELISA

Polyvinyl chloride microtitration plates were coated overnight at 4 °C with a recombinant fusion protein comprising the ECD of human IL-2R beta subunit and mouse IgG2a Fc, produced in High Five insect cells^29^, at 10 µg/mL in PBS. Coated plates were blocked with M-PBS during 30 min at RT. Recombinant fusion proteins comprising hIL-2 variants and human Fc domains were properly diluted in M-PBS and incubated on coated/blocked plates during 1 h at RT. Plates were washed with PBS-T and an anti-human IgG antibody conjugated to horseradish peroxidase (HRP), diluted in M-PBS, was added for 1 h at RT. After washing with PBS-T, peroxidase substrate solution was added. The reaction was stopped 15 min later with 10% (v/v) H_2_SO_4_. The absorbances at 490 nm were determined with a microplate reader.

### Determination of the aggregation status of recombinant proteins by size exclusion chromatography

Protein separation was performed in a TSKgelG3000SWXL column (5 μm, 300 mm × 7.8 mm), at 1 mL/min during 20 min. RHPLC Jasco 4000 high efficiency modular system was used for detection at 280 nm. Data acquisition and integration were done with the Spectra Manager platform, using the ChromNav software (version 2.01.01). A calibrator (Biorad, USA) comprising five molecules of known molecular weight (Thyroglobulin 670 kDa, IgG 158 kDa, Ovalbumin 44 kDa, Myoglobin 17 kDa, B12 1.35 kDa) was also analysed.

### Analysis of binding kinetics and affinity by surface plasmon resonance

Binding experiments were performed using the Biacore T200 instrument and the Control software 2.0.1 (GE Healthcare, USA). An amine-coupling kit was used for covalent attachment of the recombinant fusion protein comprising human beta chain ECD and mouse IgG2a Fc^29^ (at 60 µg/mL in 10 mmol/L sodium acetate buffer, pH 4) to one of the flow-cells of a CM5 biosensor chip. Bovine serum albumin was similarly immobilized on a second (blank) flow-cell. Serial dilutions of Fc-fusion proteins containing hIL-2 or hIL-2-derived mutated variants (in a range between 1 and 800 nmol/L) were injected at 15 µL/min in PBS with 0.05% Tween 20 as a running buffer. Single-cycle mode was used and the chip was regenerated with two injections of 10 mmol/L glycine buffer, pH 3, between samples. Sensorgrams were analysed using Biacore T200 evaluation software 3.0, and kinetic data were globally fitted to the 1:1 model. The quality of each experiment was judged by taking into account the curvature, baseline levels, bulk contribution, kinetic constants within the operating range of the instrument, Chi2 and U-values, as recommended by the manufacturer. At least two valid experiments were recorded for each sample.

### In silico structural analysis

The starting hIL-2 structure template was taken from Protein Data Bank (PDB ID: 1M47). Models of each mutated hIL-2 variant was constructed with the Robetta protein structure prediction and analysis server (http://robetta.bakerlab.org), using the comparative modelling method^39^. The structure of the IL-2/IL-2R beta complex was extracted from the crystal structure of the quaternary complex (PDB ID: 2B5I) with Pymol software and used as the template to construct models of the mutated IL-2 variants in complex with IL-2R beta subunit. Visualization and analysis of the structures were performed with Pymol.

Sequence propensity to form secondary structures was calculated according to three different scales (Chou-Fasman, Levitt, and Deleage-Roux) using the Expasy web server^40^. Aggregation-prone segments were predicted using the WALTZ method^18^. The above-referred 3D models of mutated IL-2 molecules with replacements at position 92 (after energy minimization with Moe using the amber force field) were used to perform coarse grained Multiplexed Replica Exchange Molecular Dynamics simulations (six replicates for each molecule) with the UNRES force field^19,41^.

### Induction of proliferation of CTLL-2 and CD25-KO CTLL-2 cells by IL-2-derived recombinant fusion proteins

Mouse CTLL-2 cell line (American Type Culture Collection, TIB-214) was grown in RPMI 1640 (Gibco, USA) supplemented with 10% heat-inactivated FBS, 2 mmol/L glutamine, and 1 μg/mL. of recombinant IL-2 no-alpha mutein (CIM, Cuba)^9^ at 37 °C under a humidified 5% CO_2_ atmosphere. Genetically modified CD25-KO CTLL-2 cells were generated by transduction of CTLL-2 cells with lentiviral particles encoding a sgRNA sequence which targets exon 2 of the mouse IL-2R alpha subunit gene. Lentiviruses were assembled by adherent HEK-293T cells co-transfected with lentiCRISPRv2 neo (Addgene, USA) containing the suitable targeting insert (cloned through BsmBI restriction site) and auxiliary plasmids pLPI, pLPII and pLP/VSV-G (Invitrogen, USA). After transduction, clones were isolated by limiting dilution and tested by flow cytometry with an antibody against mouse CD25 conjugated to APC, in order to confirm the absence of cell surface CD25 (Supplementary Fig. 14).

CTLL-2 (wt or KO) cells were harvested by centrifugation and washed three times with RPMI. 10^4^ cells per well in a 96 well plate were grown during 48 h in the presence of serial dilutions of each recombinant fusion protein in RPMI. Alamar blue dye (Invitrogen, USA) was subsequently added (20 μL/well), and plates were incubated during 18 h in the above described conditions. The absorbances at 540 and 630 nm were determined with a microplate reader, and the proliferation curves were constructed by plotting the values of the differences between absorbances at both wavelengths as a function of fusion protein concentrations.

### In vitro STAT5 phosphorylation induced by IL-2-derived fusion proteins

CTLL-2 (wt or KO) cells were harvested by centrifugation, washed three times with RPMI and deprived of IL-2 no-α mutein during 4 h. 2 × 10^5^ cells were incubated with serial dilutions of each recombinant fusion protein in RPMI during 40 min, fixed with 15 % (v/v) formaldehyde during 10 min at RT, permeabilized with cold methanol and stored at −20 °C prior to staining. Cells were subsequently washed twice with FACS buffer (2% BSA in PBS), and incubated with an APC-conjugated specific anti-human/mouse pSTAT5 antibody (Invitrogen, USA) for 30 min at 4 °C. Cell fluorescence was monitored using a Gallios flow cytometer (Beckman-Coulter, USA). Curves of mean fluorescence intensity as a function of fusion protein concentrations were constructed.

### In vitro induction of proliferation of purified CD8+T cells by IL-2-derived fusion proteins

Single-cell suspensions were obtained from inguinal and mesenteric lymph nodes from healthy C57BL/6 naïve mice (6–12 weeks old, 18–20 g) kept at the Instituto de Medicina Molecular, Faculdade de Medicina, Universidade de Lisboa. CD8+ cells were isolated using the CD8+ isolation kit from Miltenyi Biotec (USA). Cells were CTV-labeled and stimulated in vitro with 5 µg/mL of recombinant Fc-fusion proteins containing hIL-2-derived beta super-binders from phage libraries. Cells treated with similarly formatted H9 superkine- and hIL-2-containing fusion proteins were included as references. Non-stimulated cells were analyzed as negative control (basal proliferation). For flow cytometry analysis, dead cells were excluded with live/dead fixable aqua dye (Thermo Fisher Scientific, USA), and fluorescence dilution was determined on a Beckton Dickinson LSR Fortessa flow cytometer. Acquired data were analyzed with FlowJo software (Tree Star).

### In vivo expansion of T cell populations following injection of IL-2-derived fusion proteins

Six groups of five female healthy C57BL/6 mice (12–18 weeks of age, 18–20 g) each, provided by the National Center for Laboratory Animals Production (CENPALAB, Cuba), received an intraperitoneal injection of 5.6 µg of each fusion protein (including a modified IgG1 (LALA) Fc domain) once per day, during four days. An additional control group received the same regime of PBS injections. 24 h after the last injection (day 5), animals were sacrificed, and their spleens were collected, weighed and macerated. Total splenocytes were counted in a Neubauer chamber. Cells were further analysed by flow cytometry (see below).

Mice experiments were performed according to the guidelines of the International Laboratory Animals Research, using standardized procedures at the Center of Molecular Immunology (CIM), Cuba. 35 mice were used in total (five animals/group). Mice were allocated to each group by randomization using the random number table. There were five mice/cage. Before the experiments, mice were kept for seven days at CIM animal care unit (acclimatization period). The animals were maintained all the time at CIM animal care unit at 20–25 °C, 60 ± 5% relative humidity, with light/darkness cycles of 12 h. Food and water were administered *ad libitum*.

The following fluorochrome-conjugated mAbs and intracellular staining sets (eBisocience, USA) were used for flow cytometry analysis: FITC-conjugated anti-CD4, PE-conjugated anti-CD122, FITC-conjugated anti-CD8, PE-conjugated anti-Foxp3, PerCP-Cy5.5-conjugated anti-CD44, APC-conjugated anti-CD25 and PE-Cy7-conjugated anti-Ki67, Foxp3 staining buffer set. Labelled cells were analysed using Gallios cytometer (Beckman-Coulter, USA) and analysis was performed with FlowJo software.

### Determination of the anti-tumour effects of IL-2-derived fusion proteins

Mouse B16F0 melanoma (American Type Culture Collection, CRL-6322) and CT-26 colon carcinoma cells (American Type Culture Collection,CRL-2638) were grown in DMEM F12 (Life Technologies, USA) supplemented with 10% heat-inactivated FBS, at 37 °C under a humidified 5% CO_2_ atmosphere. Cells were harvested using trypsin/EDTA and suspended in PBS for in vivo experiments.

Seven groups of seven female healthy C57BL/6 mice (12–18 weeks of age, 18–20 g) each, provided by the National Center for Laboratory Animals Production (CENPALAB, Cuba) were inoculated intravenously (via tail vein) with 10^5^ MB16F0 melanoma cells on the first day. Each group subsequently received 5.6 µg of one of the six fusion proteins containing a modified IgG1 Fc(LALA) domain (4 h after cell inoculation). Treatment with the fusion proteins was repeated once per day during four days. The control group received the same regime of PBS injections. At day 21 animals were sacrificed, and lungs were extracted, weighed and embedded in Bouin’s solution for counting the number of metastatic nodules.

Seven groups of five female healthy BALB/c mice (12–18 weeks of age, 18–20 g) each, provided by the National Center for Laboratory Animals Production (CENPALAB, Cuba), were inoculated subcutaneously with 10^5^ CT26 cells in the left flank. After 4 h, animals from each group received 5.6 µg of one of the fusion proteins described above. Treatment with the fusion proteins was repeated once per day during four days. The control group received the same regime of PBS injections. After tumours became palpable, their length and width were measured every other day until one tumor side reached 18 mm. Tumor volume was estimated using the formula: vol = (length × width^2^)/2.

Mice acclimatization, maintenance, and experiments, were performed according to the guidelines of the International Laboratory Animals Research, using standardized procedures at CIM, as described in the previous section.

### Statistics and reproducibility

Graph Pad Prism 7.04 software was used to construct graphs and analyse data.

Two replicates of each phage preparation were used to assess binding activity in ELISA. Two replicates of each protein concentration were also used to titrate the activity of recombinant proteins in ELISA, proliferation and phosphorylation assays. The results were confirmed through additional experiments with a second preparation of every phage/recombinant protein. At least two batches of each recombinant protein were analysed by SEC to determine its typical aggregation profile. All the recombinant proteins to be compared in the same experiment (by ELISA, SEC or in cell-based assays) were produced in parallel to reduce the influence of process variability in protein properties.

The normal distribution of measurements within each group of animals was assessed using the Shapiro-Wilk test. One-way ANOVA followed by a Tukey multiple-comparison test (*p* < 0,05) was used for comparisons between groups in the experiments measuring the expansion of immune cell populations and anti-metastatic effect (MB16F0 model). In the case of data collected in the CT26-based experimental setting, a two-way ANOVA was performed, followed by a Tukey post-test (*p* < 0.05).

### Reporting summary

Further information on research design is available in the Nature Portfolio Reporting Summary linked to this article.

### Supplementary information

Supplementary Information

Reporting Summary

## Data Availability

All data generated and analysed during the current study are included in the published article and its supplementary information file. Source data for all graphs are deposited at Dryad (10.5061/dryad.kh18932c8). Any additional information is available from the corresponding author upon request.
