# Supplementary material for: Molecular reshaping of phage-displayed Interleukin-2 at beta chain receptor interface to obtain potent super-agonists with improved developability profiles
Source: Commun Biol. 2023 Aug 9;6:828. doi: 10.1038/s42003-023-05188-0 (PMC10412584; doi:10.1038/s42003-023-05188-0)
Supplement: Supplementary file 1 — Supplementary Information [file 42003_2023_5188_MOESM1_ESM.pdf]

Supplementary information

Title:

Molecular reshaping of phage-displayed Interleukin-2 at beta chain receptor interface to obtain potent super-agonists with improved developability profiles

Authors: Gertrudis Rojas[1*], Ernesto Relova-Hernández[1], Annia Pérez-Riverón[1], Camila Castro-Martínez[1], Osmany Diaz-Bravo[1], Yanelys Cabrera Infante[1], Tania Gómez[1], Joaquín Solozábal[1], Ana Beatriz DíazBravo[1], Maren Schubert[2,] Marlies Becker[2], Beatriz Pérez-Massón[1], Dayana Pérez-Martínez[1], Rydell Alvarez-Arzola[1], Osmany Guirola[3], Glay Chinea[3], Luis Graca[4], Stefan Dübel[2], Kalet León[1], Tania Carmenate[1]

Author affiliations:

[1]Center of Molecular Immunology, calle 216 esq 15, apartado 16040, Atabey, Playa, CP 11300, La Habana, Cuba

[2]Technische Universität Braunschweig, Institute of Biochemistry, Biotechnology and Bioinformatics, Department of Biotechnology, Spielmannstraße 7, 38106 Braunschweig, Germany

[3] Center of Genetic Engineering and Biotechnology, Ave 31 e/ 158 y 190, apartado 6162, Playa, CP 11300, La Habana, Cuba

[4]Instituto de Medicina Molecular João Lobo Antunes, Faculdade de Medicina da Universidade de Lisboa, Centro Académico de Medicina de Lisboa, Lisbon Portugal

*Correspondence should be addressed to G.R. (grojas@cim.sld.cu)

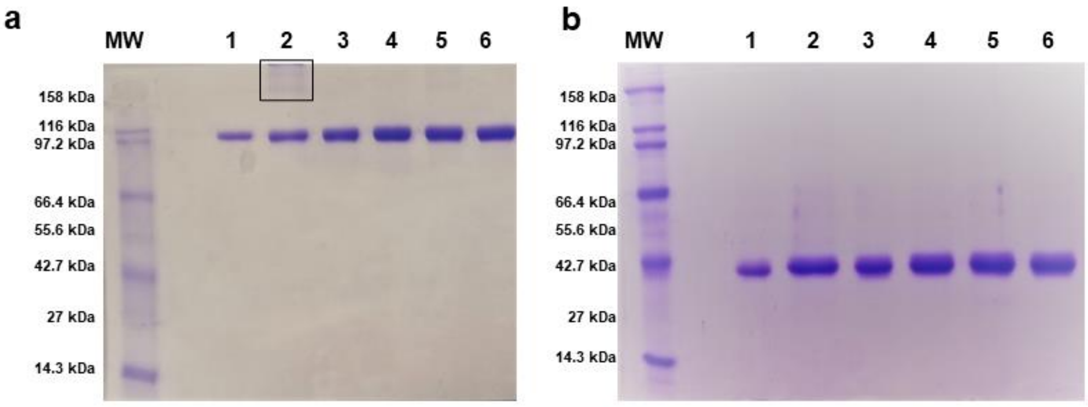

MW: molecular mass ladder

1: IL-2(K35E)/Fc

2: H9(K35E)/Fc

3: $_{81}$DPQDLIE$_{87}$(K35E)/Fc

4: $_{81}$VPEDLIE$_{87}$(K35E)/Fc

5: $_{81}$DPQDLIE$_{87}$+I92L(K35E)/Fc

6: $_{81}$VPEDLIE$_{87}$+I92L(K35E)/Fc

Supplementary Figure 1. SDS/PAGE characterization of recombinant Fc-fusion proteins comprising mutated hIL-2 variants derived from S1 phage library screening. Recombinant proteins comprising the hIL-2 variants, fused to a human IgG1 Fc region, were produced by transient transfection of HEK-293T cells adapted to grow in suspension, and purified by protein A affinity chromatography. Control fusion proteins derived from IL-2 and H9 superkine were also obtained. All the recombinant proteins included the replacement K35E. Three μg of each purified protein were applied to a 12% polyacrylamide gel, under either non-reducing (a) or reducing (b) conditions. Denaturation-resistant aggregates present in H9(K35E)/Fc control protein are enclosed in the square.

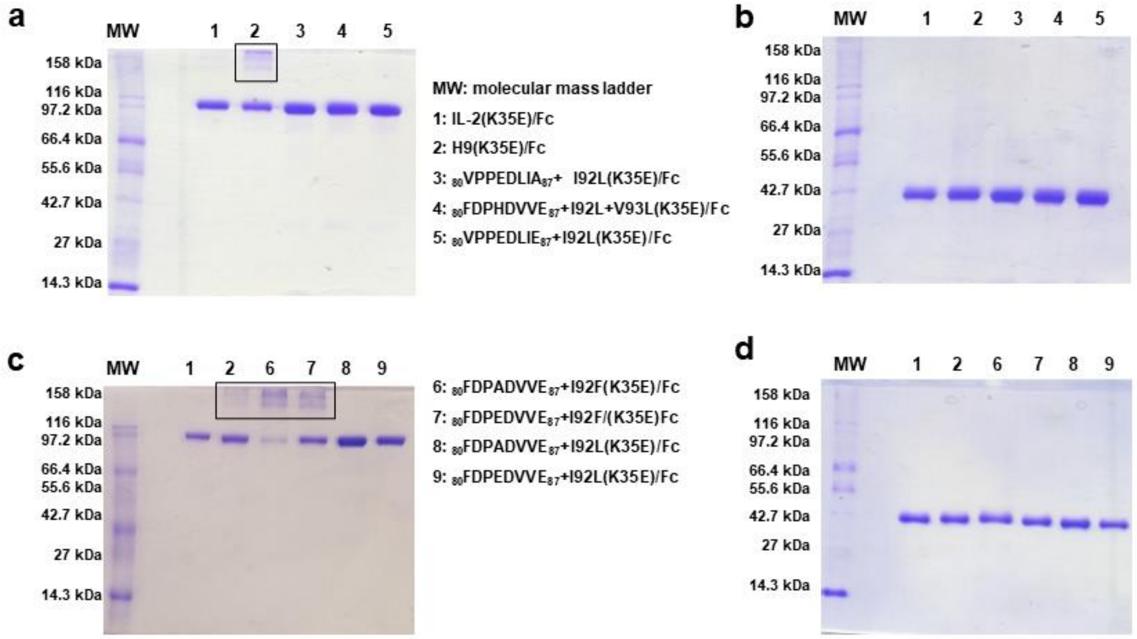

Supplementary Figure 2. SDS/PAGE characterization of recombinant Fc-fusion proteins comprising mutated IL-2 variants derived from S2 phage library screening. Recombinant proteins comprising the hIL-2 variants, fused to a human IgG1 Fc region, were produced by transient transfection of HEK-293T cells adapted to grow in suspension, and purified by protein A affinity chromatography. Control fusion proteins derived from IL-2 and H9 superkine, were also obtained. Three μg of each purified protein were applied to a 12% polyacrylamide gel, under either non-reducing (a,c) or reducing (b,d) conditions. Denaturation-resistant aggregates present in I92F-containing proteins are enclosed in squares.

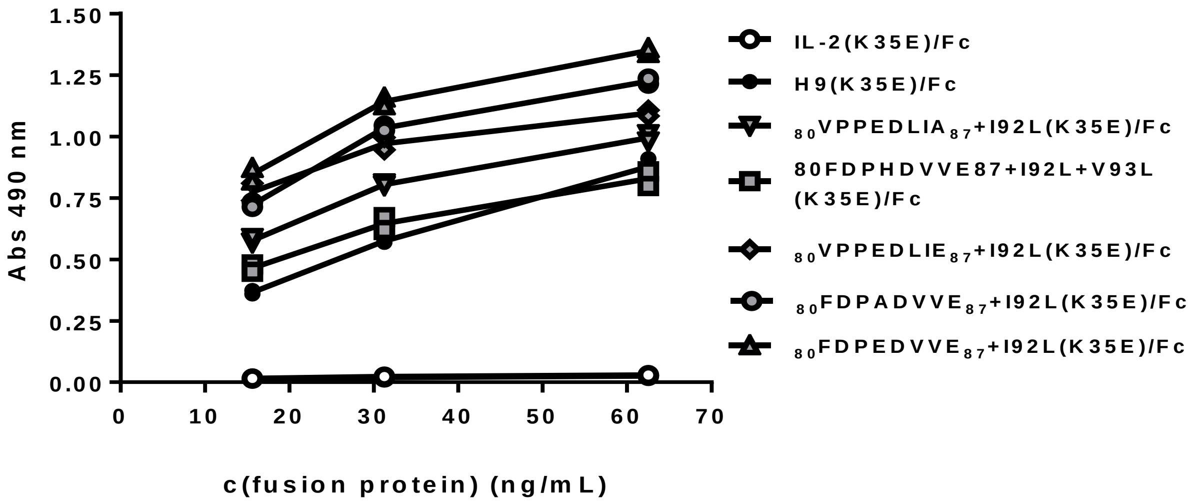

Supplementary Figure 3. Characterization of the binding properties of recombinant fusion proteins containing IL-2 variants designed on the bases of S2 phage library screening. Recombinant proteins comprising the hIL-2 variants, fused to a human IgG1 Fc region, were produced by transient transfection of HEK-293T cells adapted to grow in suspension, and purified by protein A affinity chromatography. Control fusion proteins derived from IL-2 and H9 superkine, were also obtained. The reactivity of the purified proteins was titrated by ELISA on polyvinyl chloride microtitration plates coated with a recombinant fusion protein comprising human IL-2R beta subunit extracellular domain and mouse IgG2a Fc. Bound proteins were detected with an anti-human IgG antibody conjugated to horseradish peroxidase. Symbols represent the absorbance values of two replicates of each protein concentration.

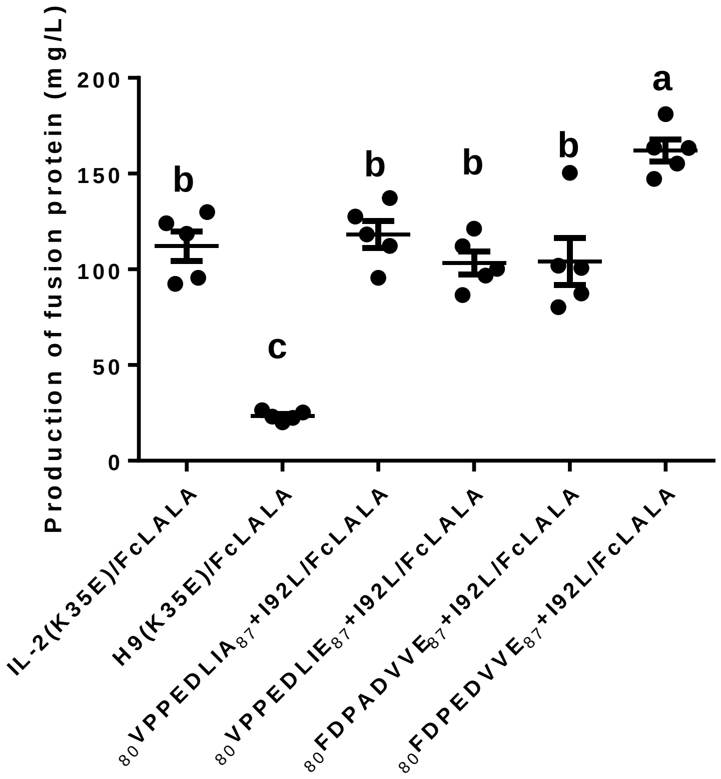

Supplementary Figure 4. Production of recombinant fusion proteins comprising hIL-2 mutated variants and human IgG1 Fc domain (including LALA mutations) by stably transduced clones. HEK-293 cells were transduced with lentiviral vectors coding for the fusion proteins which contain the new beta super-binders obtained through directed evolution. Control fusion proteins derived from IL-2 and H9 superkine, were also included. The resulting oligoclonal cell lines were cloned by limiting dilution, and secretion of recombinant proteins was tested by ELISA. The best producer clones were adapted to grow in suspension in serum-free media. Their production levels are shown in the graph. Each dot corresponds to a different clone. Lines represent mean values, while error bars indicate SD, within the set of clones producing the same variant. One-way ANOVA followed by a Tukey multiple-comparison test ($p<0,05$) was used for comparisons between groups. Different letters mean significant differences between sets of clones.

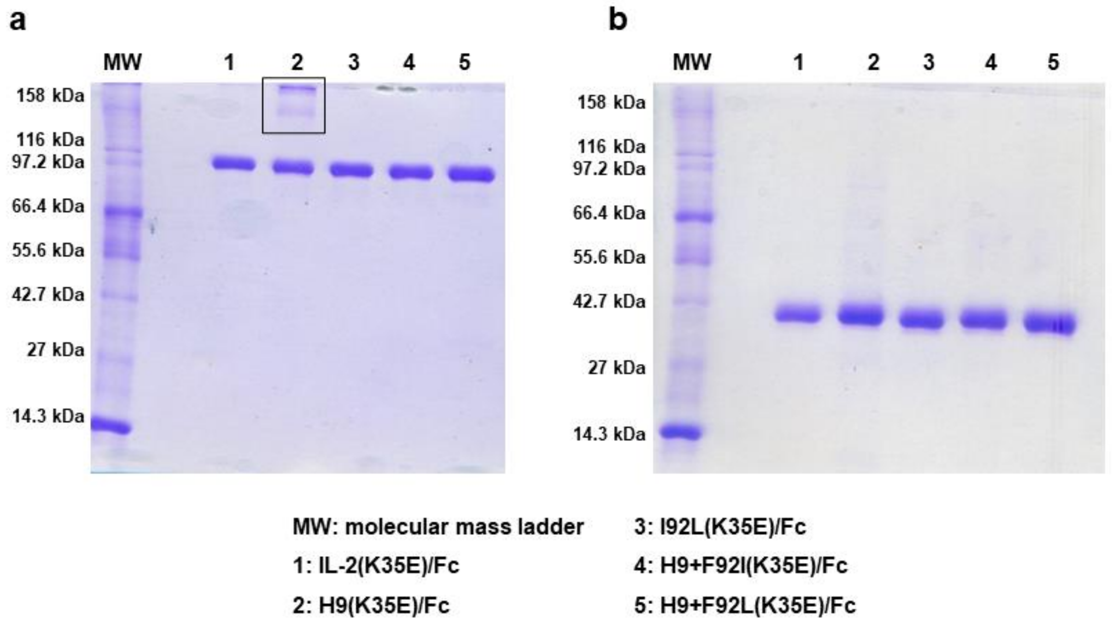

Supplementary Figure 5. SDS/PAGE characterization of recombinant Fc-fusion proteins comprising mutated IL-2 variants with changes at position 92. hIgG1 Fc-fusion recombinant proteins based on human IL-2 and its mutated I92L variant, as well as on H9 superkine and two H9-derived versions containing the replacements F92I and F92L, were produced by transient transfection of HEK-293T cells adapted to grow in suspension, and purified by protein A affinity chromatography. Three μg of each purified protein were applied to a 12% polyacrylamide gel, under either non-reducing (a) or reducing (b) conditions. Denaturation-resistant aggregates present in the superkine-containing protein appear enclosed in the square.

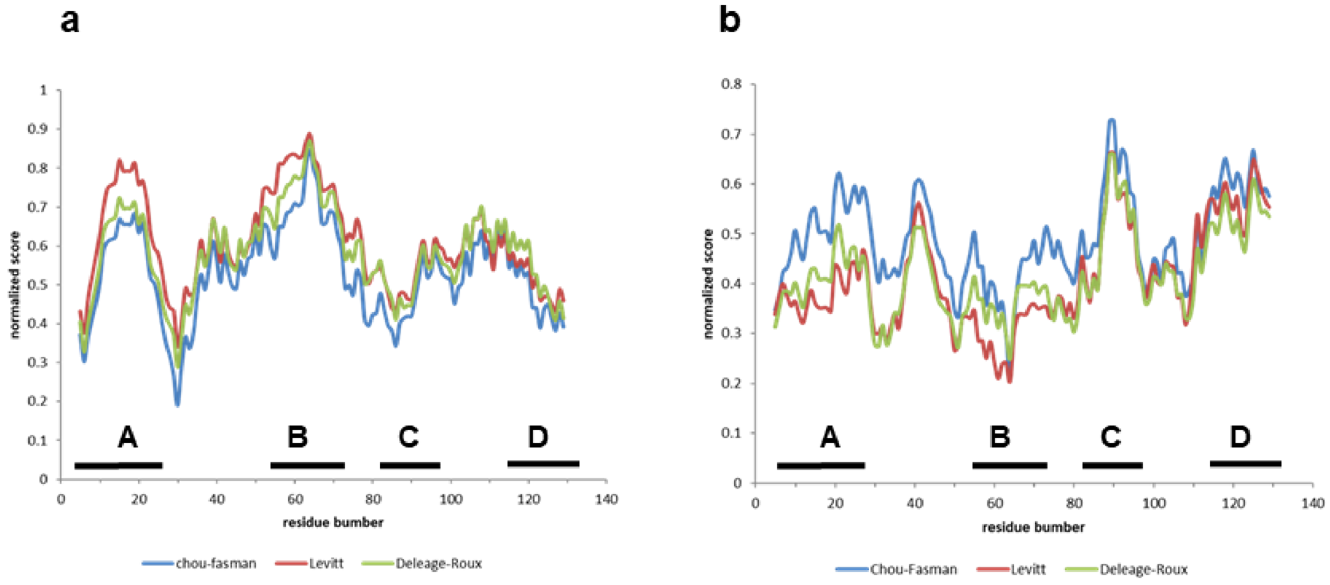

Supplementary Figure 6. Propensities to adopt secondary structures along the primary sequence of human IL-2. Relative sequence propensities to form alpha-helical (a) and beta-sheet (b) structures were calculated according to three different scales (Chou-Fasman, Levitt, and Deleage-Roux, represented with different colors) using the Expasy web server. Black lines indicate the location of the four alpha helices present in fully folded IL-2 (named A, B, C and D).

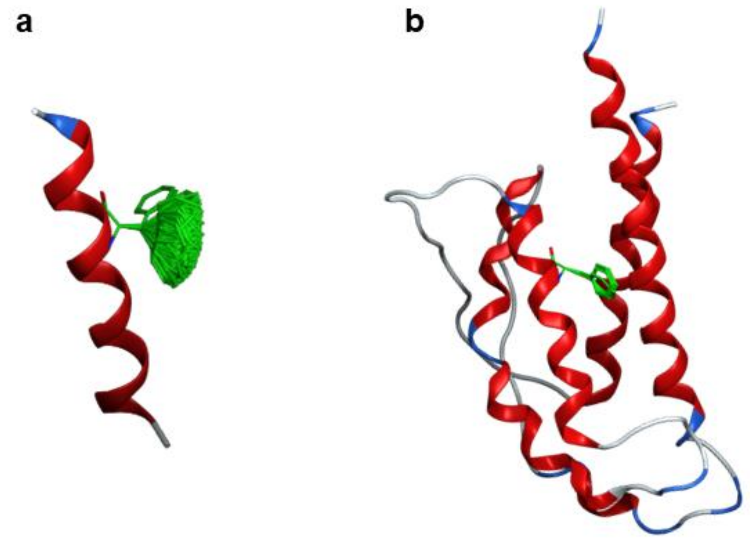

Supplementary Figure 7. Possible rotamers of F92. 3D structures of the isolated alpha-helix C (a) and the whole mutated hIL-2 with the I92F replacement (b) are represented as cartoons. Alpha-helical segments are shown in red, while loops appear in blue and white. The side chain of the residue F92 (diverse rotamers) is represented with green sticks. Whereas multiple rotamers of F92 could exist in the isolated alpha-helix C (a), only a few of them can be accommodated within the four alpha-helix bundle that forms fully folded hIL-2 (b), indicating an entropy loss associated with the replacement I92F.

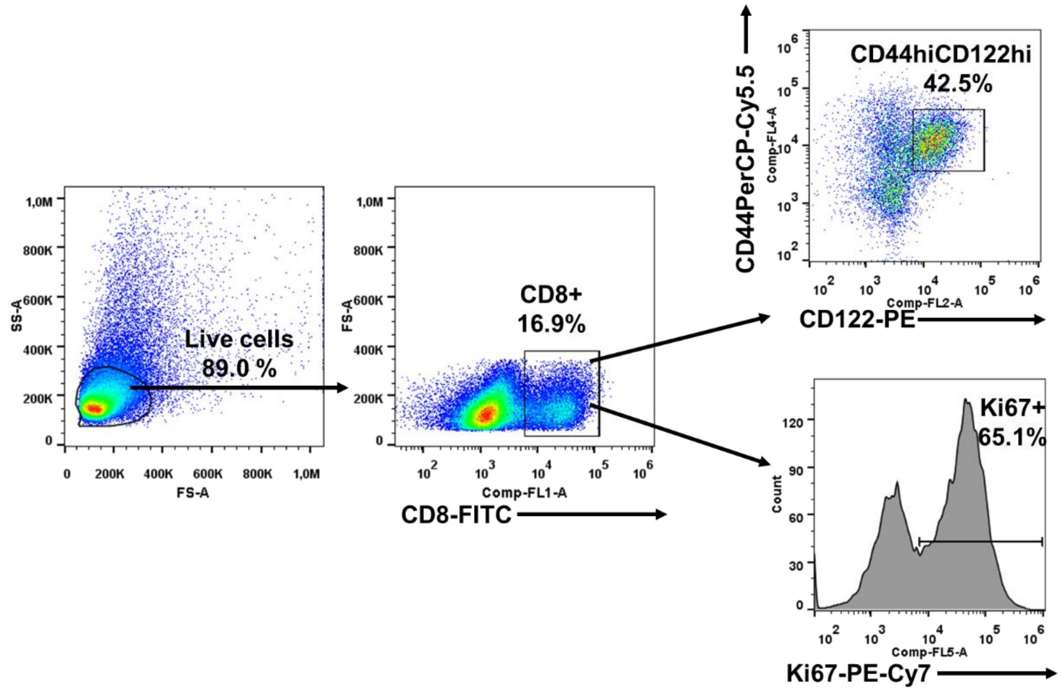

Supplementary Figure 8. Gating strategy for the analysis of effector T cells from mice. Live cells were selected first from FSC *vs* SSC graph, then were analysed for CD8 expression (CD8-FITC *vs* FSC). Finally, positive cells were analysed for activation (CD122-PE *vs* CD44-PerCP-Cy5.5) or proliferation markers (histogram *vs* Ki67-PE-Cy7). The procedure is illustrated with one sample of splenocytes.

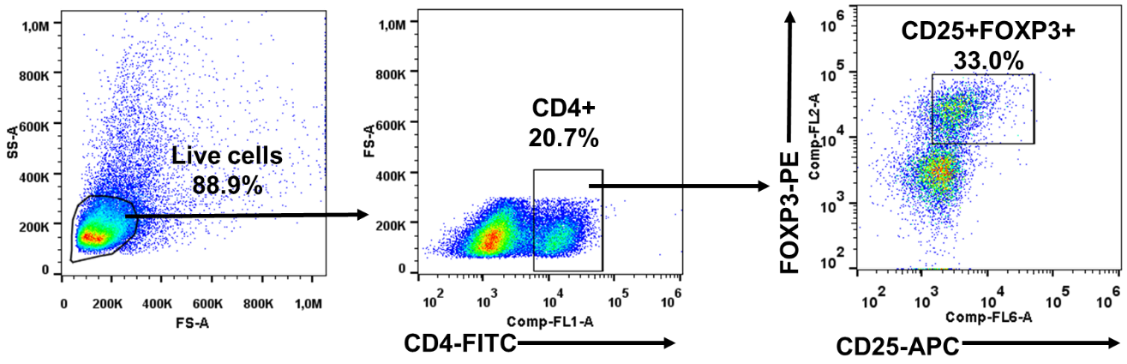

Supplementary Figure 9. Gating strategy for the analysis of T regulatory cells from mice. Live cells were selected first from FSC *vs* SSC graph, then were analyzed for CD4 expression (CD4-FITC *vs* FSC). Positive cells were finally tested for Treg markers (CD25-APC *vs* FOXP3-PE). The procedure is illustrated with one sample of splenocytes.

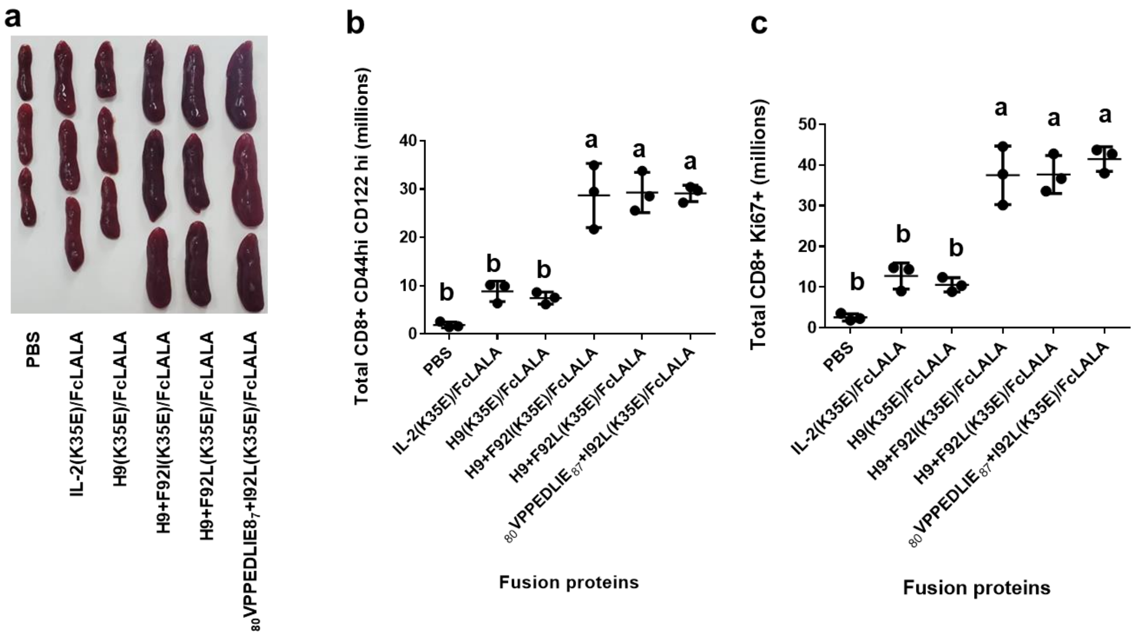

Supplementary Figure 10. *In vivo* expansion of effector T cells induced by recombinant fusion proteins comprising mutated H9-derived variants with changes at position 92. Three groups of three mice each received daily injections (during four days) of recombinant proteins comprising H9 superkine fused to human Fc(LALA) and similarly formatted molecules with modified H9 versions containing the replacements F92I and F92L. Two additional groups were treated with the fusion proteins based on either hIL-2 or the already characterized super-agonist variant $_{80}$VPPEDLIE$_{87}$+I92L(K35E)/Fc(LALA), Mice from a control group received PBS injections. All the animals were sacrificed at the fifth day, and their spleens were collected and macerated for cell characterization by flow cytometry. Spleen enlargement induced by treatment with fusion proteins is shown (a). The expansion of the effector T cell population (CD8+CD44hiCD122hi) induced by fusion proteins, was assessed by flow cytometry (b). The increase in proliferating effector cells (CD8+Ki67+), also assessed by flow cytometry, is shown in c. Lines represent mean values, while error bars indicate SD within each group. One-way ANOVA followed by a Tukey multiple-comparison test ($p<0,05$) was used for comparisons between groups. Different letters mean significant differences between groups.

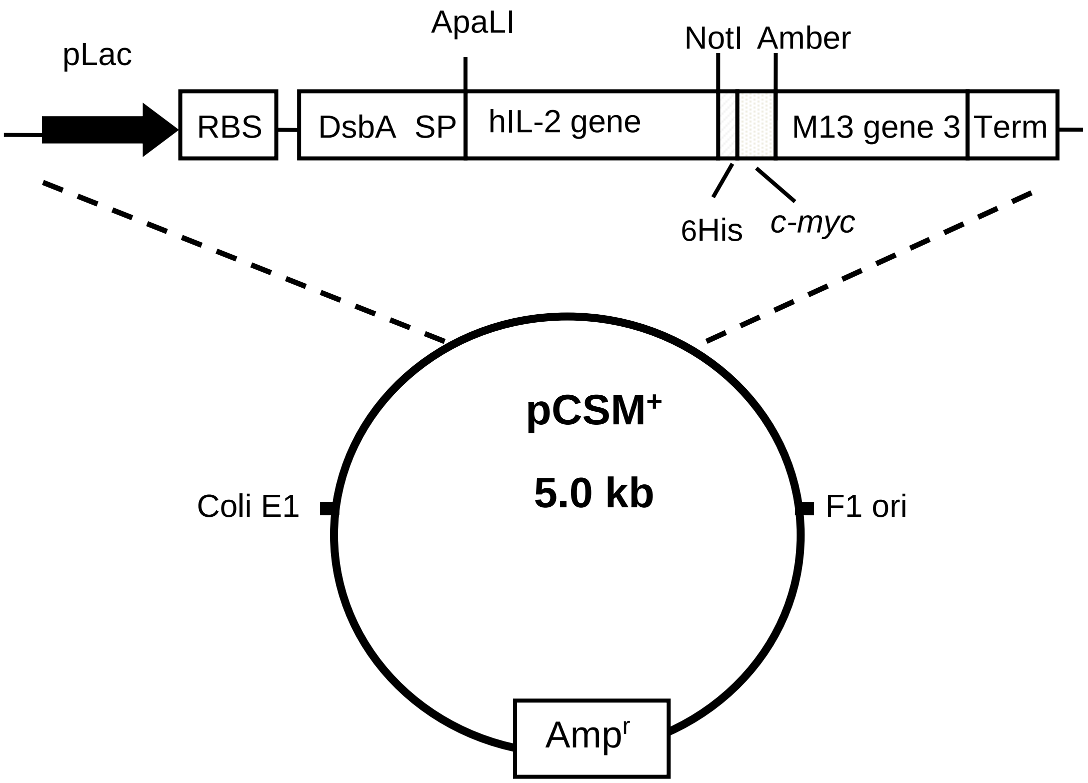

Supplementary Figure 11. Schematic representation of the phagemid vector pCSM containing the hIL-2 gene. The vector includes DNA sequences coding for *DsbA* signal peptide (MKKIWLALAGLVLAFSASA), human IL-2, and hexahistidine and *c-myc* tags. Additional elements are shown: lac promoter, ribosome binding site, amber stop codon, ApaLI and NotI restriction sites, M13 gene 3, transcription terminator sequence, E.coli and phage replication origins, and ampicillin resistance gene.

a) **5'GATGCCGTTCAGAATCATCTGCAGGTCCAGCAGCAGATGTTCCAGTTGCAGCTGGGTTTTTTTGGT3'**

b) **5'TTCGCTGCCTTTCAGTTCCAGCACAATCACGTTGATGTTGCTAATCAGATCACGCGGACGCAGATGAAAGTTTTT3'**

Supplementary Figure 12. Spiked antisense mutagenic oligonucleotides used for soft-randomization of hIL-2 interface with IL-2 receptor beta chain. Soft-randomized positions within segments 12-23 (a) and 81-95 (b) of hIL-2 are shaded in grey. Each of them contained 90% of the original nucleotide (written in the sequences) and 10% of the mix of the three remaining nucleotides.

a) **5'AATCACGTTGATGTT<u>MNN</u>AATCAG<u>MNNMNNCGSMNN</u>CAGATGAAAGTTTTT3'**

b) **5'GCCTTTCAGTTCCAG<u>MANMAN</u>CACGTT<u>MAN</u>GTT<u>MNNMANMANMNNMNNCGSMNNMAN</u>ATGAAAGTTTTTGCT3'**

Supplementary Figure 13. Degenerate antisense mutagenic oligonucleotides used for diversification of selected positions within hIL-2 interface with IL-2 receptor beta chain. Diversified positions within secondary libraries S1 (a) and S2 (b) are underlined. hIL-2 positions 81, 83, 84 and 87 were fully randomized by introduction of NNK codons coding for the mixture of the 20 amino acids. P82 was replaced by a mix of Pro and Ala (encoded by the triplet SCG). Positions 80, 85, 86, 89, 92 and 93 were occupied by a mixture of hydrophobic residues (Ile/Leu/Met/Phe/Val), encoded by the codon NTK.

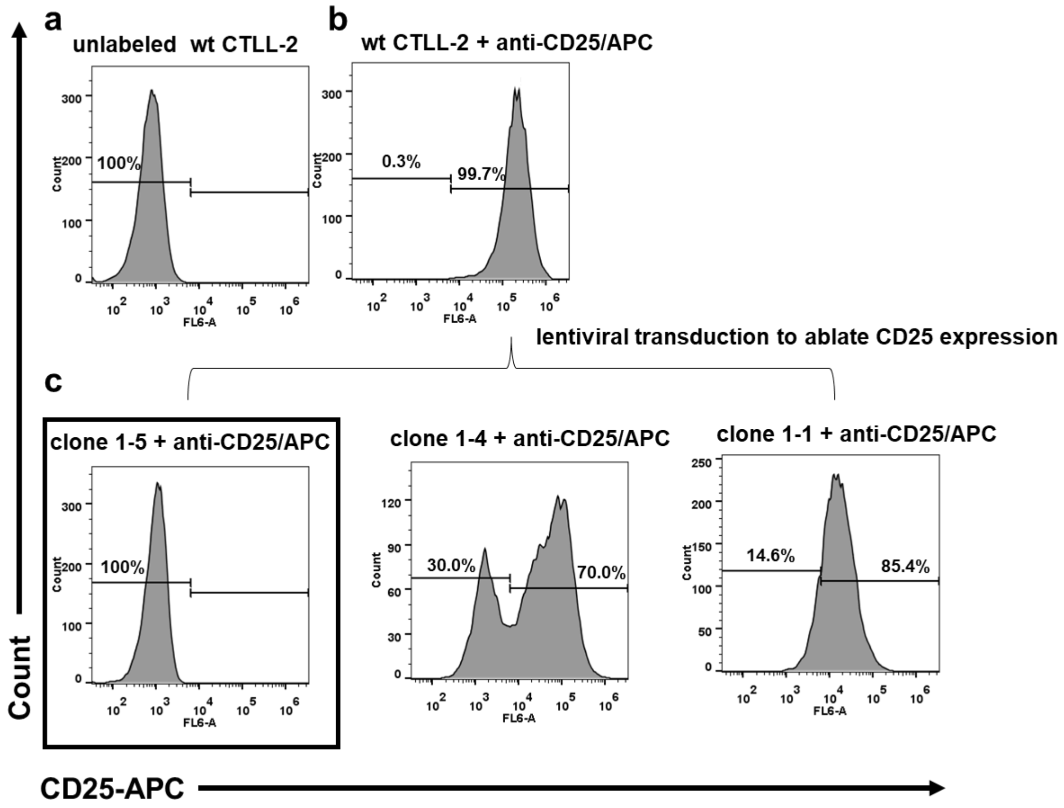

Supplementary Figure 14. Identification of CD25-KO CTLL-2 clones. CTLL-2 cells were transduced with lentiviral particles encoding a sgRNA sequence which targets exon 2 of mouse IL-2R alpha subunit gene. After transduction, clones were isolated by limiting dilution and tested by flow cytometry with an antibody against mouse CD25 conjugated to APC, in order to confirm the absence of cell surface CD25. Control unlabeled CTLL-2 cells are shown in a. CD25 staining of wt CTLL-2 cells is represented in b. Percentages indicate the abundance of negative (left) and positive cells (right) in each sample. Panel c illustrates the diversity of clones arising from lentiviral transduction. Clone 1-5 (enclosed in the square) exhibited full ablation of CD25 expression and was selected as a source of CD25-KO cells.
