# Supplementary material for: Molecular reshaping of phage-displayed Interleukin-2 at beta chain receptor interface to obtain potent super-agonists with improved developability profiles
Source: Commun Biol. 2023 Aug 9;6:828. doi: 10.1038/s42003-023-05188-0 (PMC10412584; doi:10.1038/s42003-023-05188-0)
Supplement: Supplementary file 2 — Reporting Summary [file 42003_2023_5188_MOESM2_ESM.pdf]

nature portfolio

Corresponding author(s): Gertrudis Rojas

Last updated by author(s): 15.07.2023

Reporting Summary

Nature Portfolio wishes to improve the reproducibility of the work that we publish. This form provides structure for consistency and transparency in reporting. For further information on Nature Portfolio policies, see our Editorial Policies and the Editorial Policy Checklist.

Statistics

For all statistical analyses, confirm that the following items are present in the figure legend, table legend, main text, or Methods section.

n/a	Confirmed	
☐	☒	The exact sample size (*n*) for each experimental group/condition, given as a discrete number and unit of measurement
☐	☒	A statement on whether measurements were taken from distinct samples or whether the same sample was measured repeatedly
☐	☒	The statistical test(s) used AND whether they are one- or two-sided<br>*Only common tests should be described solely by name; describe more complex techniques in the Methods section.*
☒	☐	A description of all covariates tested
☐	☒	A description of any assumptions or corrections, such as tests of normality and adjustment for multiple comparisons
☐	☒	A full description of the statistical parameters including central tendency (e.g. means) or other basic estimates (e.g. regression coefficient) AND variation (e.g. standard deviation) or associated estimates of uncertainty (e.g. confidence intervals)
☐	☒	For null hypothesis testing, the test statistic (e.g. *F*, *t*, *r*) with confidence intervals, effect sizes, degrees of freedom and *P* value noted<br>*Give P values as exact values whenever suitable.*
☒	☐	For Bayesian analysis, information on the choice of priors and Markov chain Monte Carlo settings
☒	☐	For hierarchical and complex designs, identification of the appropriate level for tests and full reporting of outcomes
☒	☐	Estimates of effect sizes (e.g. Cohen's *d*, Pearson's *r*), indicating how they were calculated

Our web collection on statistics for biologists contains articles on many of the points above.

Software and code

Policy information about availability of computer code

Data collection	*Provide a description of all commercial, open source and custom code used to collect the data in this study, specifying the version used OR state that no software was used.*
Data analysis	*Provide a description of all commercial, open source and custom code used to analyse the data in this study, specifying the version used OR state that no software was used.*

For manuscripts utilizing custom algorithms or software that are central to the research but not yet described in published literature, software must be made available to editors and reviewers. We strongly encourage code deposition in a community repository (e.g. GitHub). See the Nature Portfolio guidelines for submitting code & software for further information.

Data

Policy information about availability of data

All manuscripts must include a data availability statement. This statement should provide the following information, where applicable:

- Accession codes, unique identifiers, or web links for publicly available datasets
- A description of any restrictions on data availability
- For clinical datasets or third party data, please ensure that the statement adheres to our policy

All data generated and analysed during the current study are included in the published article and its supplementary information file. Source data for all graphs are deposited at Dryad (doi:10.5061/dryad.kh18932c8). Any additional information is available from the corresponding author upon request.

Human research participants

Policy information about studies involving human research participants and Sex and Gender in Research.

Reporting on sex and gender	*Use the terms sex (biological attribute) and gender (shaped by social and cultural circumstances) carefully in order to avoid confusing both terms. Indicate if findings apply to only one sex or gender; describe whether sex and gender were considered in study design whether sex and/or gender was determined based on self-reporting or assigned and methods used. Provide in the source data disaggregated sex and gender data where this information has been collected, and consent has been obtained for sharing of individual-level data; provide overall numbers in this Reporting Summary. Please state if this information has not been collected. Report sex- and gender-based analyses where performed, justify reasons for lack of sex- and gender-based analysis.*
Population characteristics	*Describe the covariate-relevant population characteristics of the human research participants (e.g. age, genotypic information, past and current diagnosis and treatment categories). If you filled out the behavioural & social sciences study design questions and have nothing to add here, write "See above."*
Recruitment	*Describe how participants were recruited. Outline any potential self-selection bias or other biases that may be present and how these are likely to impact results.*
Ethics oversight	*Identify the organization(s) that approved the study protocol.*

Note that full information on the approval of the study protocol must also be provided in the manuscript.

Field-specific reporting

Please select the one below that is the best fit for your research. If you are not sure, read the appropriate sections before making your selection.

☒ Life sciences ☐ Behavioural & social sciences ☐ Ecological, evolutionary & environmental sciences

For a reference copy of the document with all sections, see nature.com/documents/nr-reporting-summary-flat.pdf

Life sciences study design

All studies must disclose on these points even when the disclosure is negative.

Sample size	The strictly minimal recommended number of animals for in vivo experiments is three per group (according to CICUAL regulations) taking into account animal welfare considerations, but given the individual variability of immune responses and the risk of losing animals due to treatment-unrelated causes we decided to use five animals for immune cell expansion and anti-tumor studies. In the case of anti-metastatic experiments, the number was increased to seven because of the potential handling complications associated with intravenous injection of tumor cells. These numbers are similar to those routinely used for similar experiments at our Institute.
Data exclusions	Two animals died before the completion of the anti-metastatic experiment and had to be excluded from the analysis.
Replication	For in vivo cell expansion study three experiments including measurement of all populations of interest were performed with similar results. For antitumor studies based on the MB16F0 model six experiments were performed with similar results. Two experiments were performed using the CT26 model, with similar results.
Randomization	Randomization was performed using the randon number table.
Blinding	Animal experiments were not under blinding for researchers, but the technichians doing animal manipulations and maintenance did not know the nature of the treatments (only a code for each animal group).

Reporting for specific materials, systems and methods

We require information from authors about some types of materials, experimental systems and methods used in many studies. Here, indicate whether each material, system or method listed is relevant to your study. If you are not sure if a list item applies to your research, read the appropriate section before selecting a response.

Materials & experimental systems

n/a	Involved in the study
☐	☒ Antibodies
☐	☒ Eukaryotic cell lines
☒	☐ Palaeontology and archaeology
☐	☒ Animals and other organisms
☒	☐ Clinical data
☒	☐ Dual use research of concern

Methods

n/a	Involved in the study
☒	☐ ChIP-seq
☐	☒ Flow cytometry
☒	☐ MRI-based neuroimaging

Antibodies

Antibodies used	APC-conjugated anti-pSTAT5, FITC-conjugated anti-CD4, PE-conjugated anti-CD122, FITC-conjugated anti-CD8, PE-conjugated anti-Foxp3, PerCP-Cy5.5-conjugated anti-CD44, APC-conjugated anti-CD25 and PE-Cy7-conjugated anti-Ki67 from eBioscience were used. APC-conjugated anti-CD25 was provided by Invitrogen.
Validation	All these antibodies were commercially availble for the intended use.

Eukaryotic cell lines

Policy information about cell lines and Sex and Gender in Research

Cell line source(s)	ATCC
Authentication	No further authentication was performed beyond the one reported by the supplier.
Mycoplasma contamination	Cell lines were tested to rule out mycoplasma contamination by in-house qPCR.
Commonly misidentified lines (See ICLAC register)	*Name any commonly misidentified cell lines used in the study and provide a rationale for their use.*

Animals and other research organisms

Policy information about studies involving animals; ARRIVE guidelines recommended for reporting animal research, and Sex and Gender in Research

Laboratory animals	Female healthy C57BL/6 mice (6-12 or 12-18 weeks of age, depending on the experiment). Female healthy BALB/c mice, 12-18 weeks of age.
Wild animals	No wild animals were used.
Reporting on sex	Only female animals were used. No sex-related effects are expected to influence the experimental results.
Field-collected samples	No samples were collected in the field.
Ethics oversight	Institutional Committee of Animal Care and Use (CICUAL) at the Center of Molecular Immunology (CIM) approved all the experimental protocols with living animals performed at CIM. Experiments involving animals developed at the Instituto de Medicina Molecular were approved by by ORBEA-iMM (the institutional Animal Welfare Body). Permission for animal experimentation was granted by DGAVG (Portuguese competent authority for animal protection). Animal experiments were performed in accordance with the relevant national and international guidelines for using living animals in research.

Note that full information on the approval of the study protocol must also be provided in the manuscript.

Flow Cytometry

Plots

Confirm that:

☒ The axis labels state the marker and fluorochrome used (e.g. CD4-FITC).

☒ The axis scales are clearly visible. Include numbers along axes only for bottom left plot of group (a 'group' is an analysis of identical markers).

☒ All plots are contour plots with outliers or pseudocolor plots.

☒ A numerical value for number of cells or percentage (with statistics) is provided.

Methodology

Sample preparation	After mice sacrifice, spleens were collected and macerated, red cells were lysed, and cell suspensions were filtered by 40 um filters. Filtered cell suspensions were used for flow cytometry analysis.
Instrument	Gallios cytometer (Beckman-Coulter, USA)/ Beckton Dickinson LSR Fortessa
Software	FlowJo software XV
Cell population abundance	All populations under study are above 1% of splenocytes.
Gating strategy	Cells were selected first from FSC vs SSC graph, then cells were analyzed for CD8 expression (CD8-FITC vs FSC), positive cells were analyzed for activation (CD122-PE vs CD44 PerCP Cy5.5) or proliferation markers (histogram vs Ki67-PECy7).<br>In order to analyze Tregs, cells were selected first from FSC vs SSC graph, then cells were tested for CD4 expression (CD4-FITC vs FSC), positive cells were analyzed for Treg markers (CD25-APC vs FOXP3-PE).

☒ Tick this box to confirm that a figure exemplifying the gating strategy is provided in the Supplementary Information.
